# A General Theorem and Proof for the Identification of Composed CFA Models

**DOI:** 10.1007/s11336-023-09933-6

**Published:** 2023-09-19

**Authors:** R. Maximilian Bee, Tobias Koch, Michael Eid

**Affiliations:** 1 Psychological Methods Division, Institute for Psychology, Friedrich Schiller University Jena, Am Steiger 3/Haus 1, 07743 Jena, Germany; 2 Methods and Evaluation Division, Department of Education and Psychology, Freie Universität Berlin, Habelschwerdter Allee 45, 14195 Berlin, Germany

**Keywords:** confirmatory factor analysis, identification, rank-deficient loading matrix, bifactor models, bifactor$(S-1)$ model, CT-C$(M-1)$ model

## Abstract

In this article, we present a general theorem and proof for the global identification of composed CFA models. They consist of identified submodels that are related only through covariances between their respective latent factors. Composed CFA models are frequently used in the analysis of multimethod data, longitudinal data, or multidimensional psychometric data. Firstly, our theorem enables researchers to reduce the problem of identifying the composed model to the problem of identifying the submodels and verifying the conditions given by our theorem. Secondly, we show that composed CFA models are globally identified if the primary models are reduced models such as the CT-C$(M-1)$ model or similar types of models. In contrast, composed CFA models that include non-reduced primary models can be globally underidentified for certain types of cross-model covariance assumptions. We discuss necessary and sufficient conditions for the global identification of arbitrary composed CFA models and provide a Python code to check the identification status for an illustrative example. The code we provide can be easily adapted to more complex models.

**Supplementary Information:**

The online version contains supplementary material available at 10.1007/s11336-023-09933-6.

Confirmatory factor analysis [CFA] models are ubiquitous in the social sciences. They are frequently applied in multitrait-multimethod [MT-MM] research (e.g., Eid, 2000; Eid et al., 2003; Jeon et al., 2018; Kenny, 1976; Kenny & Kashy, 1992), longitudinal or measurement of change research (e.g., Courvoisier et al., 2008; Hedeker & Gibbons, 2006; Koch et al., 2018; Little, 2013; McArdle & Nesselroade, 2014; Newsom, 2015) as well as in psychometrics (e.g., bifactor models and applications, e.g., Cai et al., 2011; Gibbons et al., 2007; Gibbons & Hedeker, 1992; Jeon et al., 2013; Rijmen, 2010), among many others.

For any statistical inference based on a CFA model to be meaningful, it is imperative that the given model is identified. A frequently encountered definition of model identification can be stated as follows. Consider a model with model-implied covariance matrix $\Sigma =\Sigma (\theta )$ as a function of model parameters $\theta$ belonging to this model’s space of permissible parameter values. The model is identified if two different parameter vectors from this model’s parameter space, say $\theta _1$ and $\theta _2$, cannot produce the same model-implied covariance matrix. In other words, the model is identified if $\Sigma (\theta _1)\ne \Sigma (\theta _2)$ whenever $\theta _1\ne \theta _2$ (Bollen, 1989; Jöreskog, 1978). If this property holds for the whole parameter space, the model is identified *everywhere*. This is in contrast to *generic* identification that allows for this property not to hold on negligible sets (more formally, sets of measure zero, see Bekker & ten Berge, 1997).

Identification is a necessary condition for the model parameters to be uniquely estimable from the data (Bollen, 1989). That is, repeated applications of the same model to the same data may result in differing conclusions if the model is not identified. Empirically, the application of underidentified models has been found to give misleading parameter estimates, which cannot be replicated in different samples (Kenny and Kashy, 1992), as well as divergent solutions and improper estimates (Geiser et al., 2014).

There is a range of well-known necessary conditions for the identification of CFA models and structural equation models [SEM] (Anderson and Rubin, 1956; Bekker et al., 1994; Bollen, 1989). Moreover, there is a great body of research establishing rules for the *local* identification of CFA models (e.g., Anderson & Rubin, 1956; Bekker, 1989; Bekker et al., 1994; Bollen, 1989; Reilly, 1995; Shapiro, 1985; Wegge, 1996). These rules determine the identification of a model for all parameters in an open neighborhood of some point of the parameter space.

Nevertheless, even if a model is locally identified everywhere, it can still be *globally* underidentified (Bollen, 1989; Reilly, 1995) and there exist neither general sufficient nor necessary conditions for the global identification for an arbitrary CFA model (Bollen, 1989; Grayson and Marsh, 1994). Hence, practitioners often resort to proving the identification of a given model algebraically. For the parameter vector $\theta$, this is done by finding the inverse function of the model-implied covariance matrix $\Sigma (\theta )$. This process can become practically infeasible if it involves solving multiple (generally nonlinear) equations simultaneously (Bollen, 1989).

However, models can be grouped by the patterns they share in their respective loading, factor, or error covariance matrices. These patterns can then be exploited to derive rules that determine the global status of identification for a whole subclass of models. Davis (1993) showed which residual covariance and loading patterns imply identification of models in which each item has factor complexity one, that is, nonzero loading for one factor only. These sufficient conditions were extended by Reilly (1995) to necessary as well as sufficient ones for the same class of models. In another work, Reilly and O’Brien (1996) stated identification conditions for the factor loadings in models where each factor has at least one item of factor complexity one.

As another example, Grayson and Marsh (1994) showed that any CFA model with diagonal error covariance matrix $\Psi$ and block-diagonal factor covariance matrix $\Phi$ with all blocks saturated is not identified if its loading matrix, $\Lambda =(\Lambda _1\vert \Lambda _2\vert \cdots )$, has one or more submatrices $\Lambda _i$ or one or more pairs of submatrices $(\Lambda _j\vert \Lambda _k)$ with linearly dependent columns. Based on this result, Grayson and Marsh (1994) provided necessary and sufficient conditions for the identification of MT-MM models.

Fang et al. (2021) building on the work of Anderson and Rubin (1956) give identification conditions for so-called two-tier bifactor models. These models consist of two submodels with diagonal factor covariance matrices, that is, uncorrelated factors. Across models, only the general factors of each submodel are allowed to correlate.

In the present article, we are equally concerned with the process of combining submodels to form a larger model, but we abstract from any specific modeling framework, such as MT-MM or bifactor models. Instead, we broaden the scope and introduce a subclass of CFA models we denote *composed models*. We give a theorem stating not only necessary but also sufficient conditions for their global identification and give recommendations on how to deal with the challenges presented by these models. The aim of this study is to supply researchers with the necessary theoretical foundations and practical guidelines that enable them to better understand why certain models behave the way they do and ensure the identification of the models they work with.

In order to delineate the notion of a composed model, we consider the following scenario. A researcher investigates the relationship between two constructs of interest in a population, expressed by two separate CFA models, which we call the *primary models*. Beyond the covariance matrices of the individual models, the researcher is particularly interested in the covariances between factors of the first and factors of the second primary model. To estimate these covariances, the two primary models must be combined into a larger model, incorporating all items from both primary models, which we refer to as the *composed model*. However, items from one primary model are assumed not to load on factors of the other primary model, such that there are no new loading parameters introduced in the composed model. Put differently, composed models can be considered general structural equation models, in which factors defined in distinct measurement models are correlated or related in a latent path model (for an overview, see Wang & Wang, 2012).

**Fig. 1:**
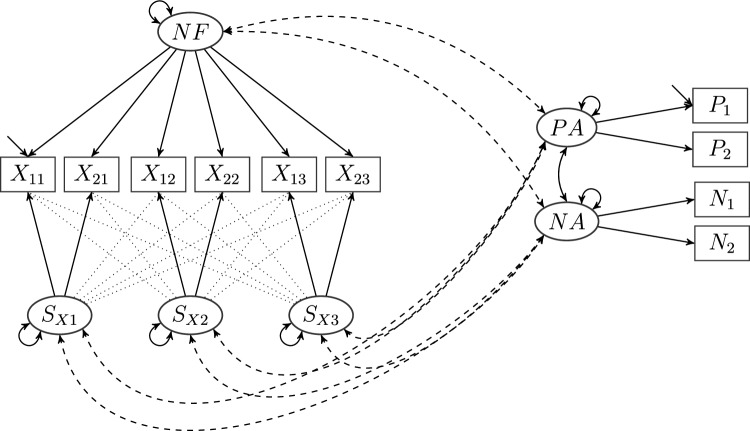
A bifactor ESEM model given by items $X_{ji}$, general factor *NF*, and specific factors $S_{Xi}$, $j\in \{1,2\}, i\in \{1,2,3\}$, on the one hand, and a two-factor model with factors *P* and *N* indicated by their respective items, $P_{i}$, $N_{i}$, $i\in \{1,2\}$, on the other. In the bifactor ESEM model, the exploratory loadings are shown with dotted lines. There are no cross-loadings, but all factors are correlated across models. Cross-model covariances are represented by dashed lines. The nomenclature is chosen to resemble the model employed by Tóth-Király et al. (2017) relating need fulfillment to positive and negative affect. Analogously to Figs. 2 and 3, the parameter labels have been omitted.

Numerous examples can be found in the literature where the factors in some CFA model are related to a single criterion such as, for example, task performance (Debusscher et al., 2017), humor (Christensen et al., 2018), or life satisfaction (Chen et al., 2012). A specific instance of this type of model is depicted in Fig. 1. It represents the approach employed by Tóth-Király et al. (2017) to relate need fulfillment, measured by a bifactor exploratory structural equation model [ESEM] (see, e.g., Marsh et al., 2014), to positive and negative affect, measured by a model with two correlated factors.

**Fig. 2:**
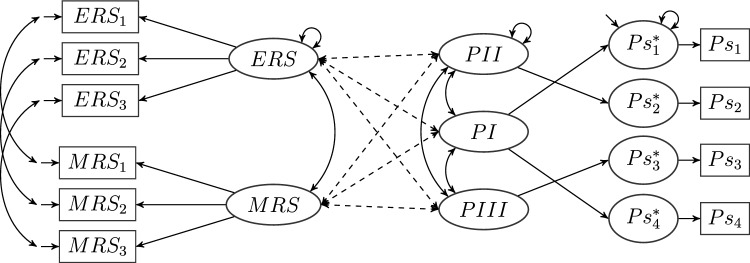
A composed model with a CT-CU model and a multiprocess IRT model as primary models. The (restricted) CT-CU model consists of factors ERS and MRS indicated by their respective items. Residual variables of items $ERS_{i}$ and $MRS_{i}$ are correlated for every *i*, $i\in \{1,2,3\}$. The multiprocess IRT model is given by factors *PI*, *PII*, *PIII*. The auxiliary latent variables $Ps_{i}^{*}$ are indicated by dichotomous manifest variables $Ps_{i}$ with unit loadings. ($Ps_{2}^{*}$ and $Ps_{3}^{*}$ are pseudo-variables and can be identified with *PII* and *PIII*, respectively.) There are no cross-loadings, but all factors are correlated across models. Cross-model covariances are represented by dashed lines. This is a simplified version of the model employed by Plieninger and Meiser (2014) to relate response styles and IRT processes, in which the factors are not simply correlated across models, but the process factors are regressed on the response style factors, and there is an additional criterion variable. Analogously to Figs. 1 and  3, the parameter labels have been omitted.

As another example, Plieninger and Meiser (2014) validated the response processes in a multiprocess item response theory [IRT] model with response style scales. With the multiprocess IRT model as the first primary model, the second one was given by analyzing the scales for extreme as well as mid response styles, forming parcels, and modeling them with a correlated traits-correlated uniqueness [CT-CU] model. In the original article, the authors used latent regression between the primary models based on theoretical considerations. Latent regression—and any other linear dependency that can be represented in a path model—is a function of the latent variances and covariances. Because we wish to abstract from any specific path model, we state our theorem in terms of these fundamental parameters they are built on. Therefore, this article lays the groundwork for the discussion of arbitrary path models in composed models, but it is beyond its scope. The correlational composed model underlying the latent regression employed by Plieninger and Meiser (2014) is shown in Fig. 2.

Our theorem covers combinations of more complex models as well such as multiconstruct growth curve models (Bollen and Curran, 2005) or latent state-trait [LST] models (e.g., Schmitt, 2000; Steyer et al., 2012). A schematic example of the former is given in Fig. 3: Two constructs, measured by two distinct growth curve models, are put in relation to another via occasion-specific correlations on the one hand and correlated intercept as well as slope factors on the other. However, in some applications of these kinds of longitudinal models the problem of autocorrelated errors might arise. In the Discussion section, we explain how correlated errors can be handled within our approach.

**Fig. 3:**
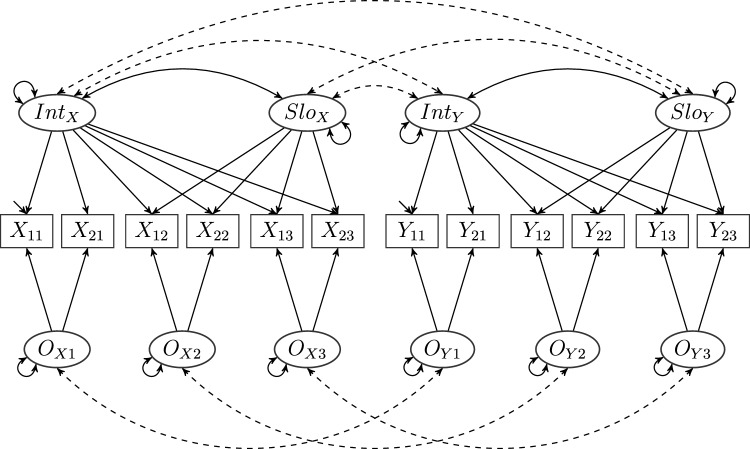
A composed model with two growth curve models (see, e.g., Bollen & Curran 2005) given by items $X_{ji}$, occasions $O_{Xi}$, intercept factor $Int_{X}$, slope factor $Slo_{X}$ as well as items $Y_{ji}$, occasions $O_{Yi}$, intercept factor $Int_{Y}$, slope factor $Slo_{Y}$, $j\in \{1,2\}$ and $i\in \{1,2,3\}$, respectively. As in Fig. 4, there are no cross-loadings. All factors are correlated across models, and cross-model covariances are represented by dashed lines. Loadings, (co-)variances, and errors have not been labeled for the sake of readability because the model is not discussed further in this article.

**Fig. 4:**
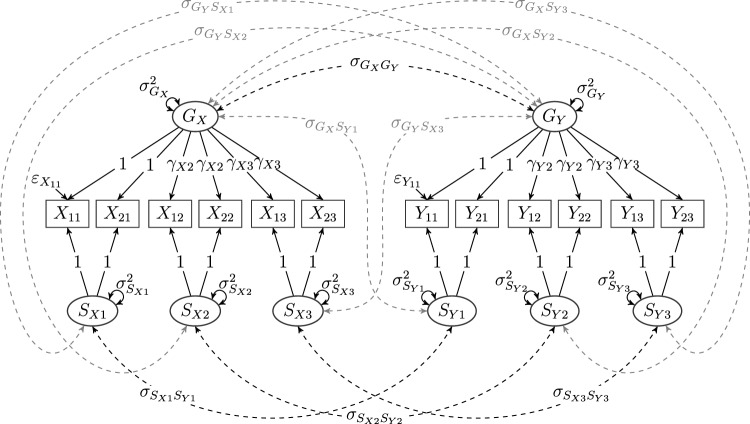
A composed model with two bifactor models as primary models, given by items $X_{ji}$, general factor $G_{X}$, and specific factors $S_{Xi}$ as well as items $Y_{ji}$, general factor $G_{Y}$, and specific factors $S_{Yi}$, $j\in \{1,2\}$ and $i\in \{1,2,3\}$, respectively. Items for the first primary (bifactor) model do not load on the second primary (bifactor) model and vice versa. Black dashed lines represent covariances on the diagonal of the cross-model factor covariance matrix $\Phi _{YX}$ as given in Eq. (9), and gray dashed lines represent off-diagonal covariances in $\Phi _{YX}$. If the covariances represented by the gray dashed lines are fixed to zero as in Eq. (10), the model is a multiconstruct bifactor model (see Koch et al., 2018).

Finally, as a another example of a composed model consisting of more complex primary models that are prominently applied in the social sciences, consider Fig. 4. The model consists of two bifactor models with one general and three specific factors each (Reise, 2012). The composed model obtained by only considering the black lines is a multiconstruct bifactor model (Eid et al., in preparation; Koch et al., 2018). Because we will reference this example throughout the article and use it to illustrate how our theorem can be applied in practice, all model parameters (i.e., loadings, variances, and covariances as well as errors) are explicitly given.

In the specific model chosen, both primary models are bifactor reformulations of hierarchical *G*-factor models (Markon, 2019; Reise, 2012). That is, their loading structures are derived from the Schmid–Leiman solution of a hierarchical model (Schmid and Leiman, 1957; Yung et al., 1999). To see what this implies, note that the loadings of the items on the specific factors need to be set to unity because there are only two items per factor. Then, because loadings on the *G*-factor in the bifactor model are equal to the product of the path coefficients along the respective paths in the hierarchical model, for each facet (i.e., for each *i*) the loadings of the items $X_{ji}$, respectively, $Y_{ji}$ on $G_{X}$, respectively, $G_{Y}$ are equal and therefore linearly dependent on the corresponding loadings on the specific factors $S_{Xi}$, respectively, $S_{Yi}$.

We emphasize, however, that the sufficient and necessary conditions we state in our theorem hold not only for the loading structure given in this example, but for all possible loading structures, such as, for example, essentially $\tau$-equivalent models (Steyer, 2015), the Green–Yang factor structure (Green and Yang, 2018), or trifactor models (Jeon et al., 2018). In fact, as the variety of the previous examples indicate, our theorem even holds not only for bifactor models but for all models that share the present form: Two primary models are combined to form a composed model, and loadings as well as error covariances are set to zero across the primary models.

However, as Eid et al. (2018) showed, even in the simple case of a construct validation study, identification issues can arise. Specifically, a composed model predicting academic achievement by all factors of a bifactor model measuring intelligence is not identified if the bifactor model has an essentially $\tau$-equivalent loading structure. That is, *although both primary models are identified*, the composed model is not. In the present article, we replicate this counterintuitive finding for a much larger class of models under which the model considered by Eid et al. (2018) can be subsumed.

The practical relevance of our theorem lies in the fact that it not only provides simple rules to determine the status of identification of a given CFA model of the type we discuss in this article, but that it is descriptive of the pattern of parameters that are underidentified and how to achieve identification algorithmically. Lastly, we conclude the article by introducing another class of CFA models we denote *reduced models* and explaining how they circumvent identification issues for the composed model. Throughout the article, we illustrate important results with the help of our introductory example.

A mathematically rigorous treatment of our results is provided in Supplementary Material such that interested readers can retrace all steps of our theorem and proofs. It gives exact definitions for the concepts presented more colloquially in the main body of the text and states the central result, its proof, and corollaries in terms of these definitions. In Supplementary Material, we also provide a more detailed definition of reduced models than is given in the main body of the text. Lastly, along with this article we provide a Python code that performs the identification analysis for the introductory example, which is easily adapted to other situations.

## Central Result

Consider a CFA model given by^1^

1 $$\begin{aligned} \Sigma _{c}=\Lambda _{c}\Phi _{c}\Lambda _{c}^{T}+\Psi _{c} \end{aligned}$$

with (block) loading matrix

2 $$\begin{aligned} \Lambda _{c}{:}{=}\left( \begin{array}{c|c} \Lambda _{1}&{}\varvec{0}\\ \hline \varvec{0}&{}\Lambda _{2} \end{array}\right) , \end{aligned}$$

(block) latent variable covariance matrix

3 $$\begin{aligned} \Phi _{c}=\left( \begin{array}{c|c} \Phi _{1}&{}\Phi _{21}^{T}\\ \hline \Phi _{21}&{}\Phi _{2} \end{array}\right) , \end{aligned}$$

and (block) error covariance matrix

4 $$\begin{aligned} \Psi _{c}=\left( \begin{array}{c|c} \Psi _{1}&{}\varvec{0}\\ \hline \varvec{0}&{}\Psi _{2} \end{array}\right) . \end{aligned}$$

We refer to this model as the *composed model*. The submatrices on the diagonals in Eqs. (2)–(4) in turn constitute CFA models given by the model equations

5 $$\begin{aligned} \Sigma _{1}=\Lambda _{1}\Phi _{1}\Lambda _{1}^{T}+\Psi _{1} \end{aligned}$$

and

6 $$\begin{aligned} \Sigma _{2}=\Lambda _{2}\Phi _{2}\Lambda _{2}^{T}+\Psi _{2}. \end{aligned}$$

The two models defined by Eqs. (5)–(6) will be the *primary models*.

The only submatrix not contained in either primary model is $\Phi _{21}$. It contains all covariances between any factor from the first primary model and any factor from the second primary model. We subsequently refer to these covariances as the *cross-model factor covariances* and to $\Phi _{21}$ as the *cross-model factor covariance matrix*.

Note that $\Phi _{21}$ might be constrained, which our theorem explicitly takes into account. In the present context, we limit ourselves to *linear* constraints that do not introduce new parameters (such as latent regression weights that can be identified once the covariances have been identified). They make up the overwhelming majority of commonly applied constraints for covariances (Bekker, 1989; Bollen, 1989), since they include, for example, setting some covariance to zero or to equal some other covariance.

The terminology introduced for our introductory example is in line with this definition: The model depicted in Fig. 4 is a composed model with two bifactor models as primary models, each with one general and three specific factors. Its primary models have respective loading matrices

7 $$\begin{aligned} \Lambda _{X}{:}{=}\begin{pmatrix} 1 &{}\quad 0 &{}\quad 0 &{}\quad 1\\ 1 &{}\quad 0 &{}\quad 0 &{}\quad 1\\ 0 &{}\quad 1 &{}\quad 0 &{}\quad \gamma _{X2}\\ 0 &{}\quad 1 &{}\quad 0 &{}\quad \gamma _{X2}\\ 0 &{}\quad 0 &{}\quad 1 &{}\quad \gamma _{X3}\\ 0 &{}\quad 0 &{}\quad 1 &{}\quad \gamma _{X3} \end{pmatrix}\quad \text {and}\quad \Lambda _{Y}{:}{=}\begin{pmatrix} 1 &{}\quad 0 &{}\quad 0 &{}\quad 1\\ 1 &{}\quad 0 &{}\quad 0 &{}\quad 1\\ 0 &{}\quad 1 &{}\quad 0 &{}\quad \gamma _{Y2}\\ 0 &{}\quad 1 &{}\quad 0 &{}\quad \gamma _{Y2}\\ 0 &{}\quad 0 &{}\quad 1 &{}\quad \gamma _{Y3}\\ 0 &{}\quad 0 &{}\quad 1 &{}\quad \gamma _{Y3} \end{pmatrix} \end{aligned}$$

as well as factor covariance matrices

8 $$\begin{aligned} \Phi _{X}{:}{=}\begin{pmatrix} \sigma _{S_{X1}}^2 &{}\quad 0 &{}\quad 0 &{}\quad 0\\ 0 &{}\quad \sigma _{S_{X2}}^2 &{}\quad 0 &{}\quad 0\\ 0 &{}\quad 0 &{}\quad \sigma _{S_{X3}}^2 &{}\quad 0\\ 0 &{}\quad 0 &{}\quad 0 &{}\quad \sigma _{G_{X}}^2 \end{pmatrix}\quad \text {and}\quad \Phi _{Y}{:}{=}\begin{pmatrix} \sigma _{S_{Y1}}^2 &{}\quad 0 &{}\quad 0 &{}\quad 0\\ 0 &{}\quad \sigma _{S_{Y2}}^2 &{}\quad 0 &{}\quad 0\\ 0 &{}\quad 0 &{}\quad \sigma _{S_{Y3}}^2 &{}\quad 0\\ 0 &{}\quad 0 &{}\quad 0 &{}\quad \sigma _{G_{Y}}^2 \end{pmatrix}. \end{aligned}$$

Lastly, all error variables are assumed to be uncorrelated, such that the error covariance matrices $\Psi _{X}$ and $\Psi _{Y}$ of both bifactor models are diagonal. The only submatrix in the composed model yet to be defined is the cross-model factor covariance matrix $\Phi _{YX}$ (i.e., $\Phi _{21}$ in the general definition—the lower-right submatrix of $\Phi _{c}$), which is given by

9 $$\begin{aligned} \Phi _{YX}{:}{=}\begin{pmatrix} \sigma _{S_{X1}S_{Y1}} &{} 0 &{} 0 &{} \sigma _{G_{X}S_{Y1}}\\ 0 &{} \sigma _{S_{X2}S_{Y2}} &{} 0 &{} \sigma _{G_{X}S_{Y2}}\\ 0 &{} 0 &{} \sigma _{S_{X3}S_{Y3}} &{} \sigma _{G_{X}S_{Y3}}\\ \sigma _{S_{X1}G_{Y}}&{} \sigma _{S_{X2}G_{Y}} &{} \sigma _{S_{X3}G_{Y}} &{} \sigma _{G_{X}G_{Y}} \end{pmatrix}. \end{aligned}$$

As bifactor models, the primary models are identified (see Steyer et al., 2015, for details). The central question thus becomes: Under what conditions is the composed model identified, assuming that both primary models are identified by themselves? The answer is provided by the following theorem.

### Theorem 1

Let the primary models be globally identified, and let all, if any, constraints on $\Phi _{21}$ be linear and not introduce new parameters. Then, the following holds.

- The composed model is [generically] globally identified if and only if $\Lambda _{1}\otimes \Lambda _{2}=\Lambda _{1}(\theta _{\Lambda _{1}})\otimes \Lambda _{2}(\theta _{\Lambda _{2}})$ is injective for [almost] all $(\theta _{\Lambda _{1}},\theta _{\Lambda _{2}})$ on the parameter space containing the cross-model covariance parameters arranged in $\Phi _{21}$. In particular, this implies the following.
- $\Lambda _{1}=\Lambda _{1}(\theta _{\Lambda _{1}})$ and $\Lambda _{2}=\Lambda _{2}(\theta _{\Lambda _{2}})$ having full rank for [almost] all $(\theta _{\Lambda _{1}},\theta _{\Lambda _{2}})$ is sufficient for the composed model to be [generically] globally identified.
- If $\Phi _{21}$ is unrestricted, $\Lambda _{1}=\Lambda _{1}(\theta _{\Lambda _{1}})$ and $\Lambda _{2}=\Lambda _{2}(\theta _{\Lambda _{2}})$ having full rank for [almost] all $(\theta _{\Lambda _{1}},\theta _{\Lambda _{2}})$ is necessary and sufficient for the composed model to be [generically] globally identified.

If the primary models are globally identified everywhere, then both variants of Items (a)–(c) hold. If the primary models are only generically globally identified, then only the generic variants of Items (a)–(c) hold.

### Proof

Theorem 1 constitutes a summary of Theorem S.3 and its corollaries in Supplementary Material. Here, we give a concise but self-contained proof with pointers to Supplementary Material (S) such that the interested reader can find a formal justification for each step.

Recall that a map $f:U\rightarrow V$ is *injective on U* if and only if $f(x)=f(y)$ implies $x=y$ for all $x,y\in U$. The *kernel* of a linear map *A*, denoted $\ker A$, is the subspace containing all vectors that are mapped to the zero vector under *A*. Lastly, the *rank* of a linear map is the dimension of its image, which, in the case of matrices, corresponds to the maximal number of linearly independent columns, respectively, rows. Relevant references can be found in the introduction to Supplementary Material.

We start by proving Item (a) in Theorem 1 [which is a restatement of Eq. (S.20)]. For any fixed parameters $(\theta _{\Lambda _{1}},\theta _{\Lambda _{2}})$, the tensor product $\Lambda _{1}\otimes \Lambda _{2}=\Lambda _{1}(\theta _{\Lambda _{1}})\otimes \Lambda _{2}(\theta _{\Lambda _{2}})$ is a vector-valued representation of the matrix-valued linear map $\Lambda _{2}\Phi _{21}\Lambda _{1}^{T}=\Lambda _{2}(\theta _{\Lambda _{2}})\Phi _{21}\Lambda _{1}^{T}(\theta _{\Lambda _{1}})$ [taken as a function of $\Phi _{21}$, see Eq. (S.24a)]. The matrix $\Lambda _{2}\Phi _{21}\Lambda _{1}^{T}$, however, is a submatrix of the model-implied covariance matrix $\Sigma _{c}$, and thus, uniquely determining the corresponding parameters is a necessary requirement for the unique determination of $\Sigma _{c}$ and thus the identification of the model [see Eq. (S.21b)].

By the definition of injectivity, if the condition given in Item (a) is fulfilled, it implies that no two sets of cross-model factor covariance parameters can result in the same matrix $\Lambda _{2}\Phi _{21}\Lambda _{1}^{T}=\Lambda _{2}(\theta _{\Lambda _{2}})\Phi _{21}\Lambda _{1}^{T}(\theta _{\Lambda _{1}})$ and thus $\Phi _{21}$ is uniquely determined. If the condition fails on a zero-measured set of parameters $(\theta _{\Lambda _{1}},\theta _{\Lambda _{2}})$, this set is zero-measured in the whole parameter space as well such that $\Phi _{21}$ is uniquely determined for almost all parameter vectors.

To complete the proof of Item (a), we observe that three crucial assumptions—that the primary models are [generically] globally identified, that items in the composed model only load on their respective primary model’s factors, and that errors are uncorrelated across models—imply that the only yet undetermined parameters introduced in the composed model are the cross-model factor covariances in $\Phi _{21}$. The unique determination of the parameters contained in $\Phi _{21}$ is thus also sufficient to identify the full model.

Because $\Lambda _{1}\otimes \Lambda _{2}=\Lambda _{1}(\theta _{\Lambda _{1}})\otimes \Lambda _{2}(\theta _{\Lambda _{2}})$ is a linear map for any fixed $(\theta _{\Lambda _{1}},\theta _{\Lambda _{2}})$, it is injective on some subspace if and only if its kernel intersected with this subspace consists solely of the zero vector (we say the subspace is *trivial*). This condition, in turn, is determined by the column ranks of $\Lambda _{1}=\Lambda _{1}(\theta _{\Lambda _{1}})$ and $\Lambda _{2}=\Lambda _{2}(\theta _{\Lambda _{2}})$ as well as the restrictions on $\Phi _{21}$ (i.e., the equations governing the corresponding subspace). Specifically, if the loading matrices have full column ranks, then their kernels are trivial. Since $\ker \Lambda _{1}\otimes \Lambda _{2}$ is composed of the kernels of either matrix [cf. Lemma S.7], it is then trivial as well. But then $\Lambda _{1}\otimes \Lambda _{2}$ is injective, giving Item (b) by application of Item (a).

On the other hand, if any of the kernels of $\Lambda _{1}$ and $\Lambda _{2}$ are non-trivial and $\Phi _{21}$ is unrestricted, then $\ker \Lambda _{1}\otimes \Lambda _{2}$ cannot be trivial either and therefore $\Lambda _{1}\otimes \Lambda _{2}$ cannot be injective. Again, by Item (a), the model is not identified and we obtain Item (c), which concludes the proof. $\square$

To elaborate, Item (b) in Theorem 1 states that working with primary models that have full-rank loading matrices (and therefore linearly independent columns) for [almost] all loading parameter values ensures identification of the composed model, no matter how the covariances in $\Phi _{21}$ have been linearly restricted, if at all. Conversely, and somewhat surprisingly, from Item (c) we learn that even given the assumption that both primary models are identified, if there are no restrictions on the cross-model factor covariances in $\Phi _{21}$, then for the composed model to be [generically] identified, both loading matrices $\Lambda _{1}$ and $\Lambda _{2}$ *must* have full rank for [almost] all loading parameter values.

However, in applied research, rank-deficient loading matrices of the primary models often arise from theoretical reasons and thus cannot be modified without modifying the underlying theory. Instead, researchers must restrict the cross-model covariances to obtain an identified model. As can be seen from rearranging the product $\Lambda _{2}\Phi _{21}\Lambda _{1}^{T}=\Lambda _{2}(\Lambda _{1}\Phi _{21}^{T})^{T}$, $\Lambda _{2}$ acts on the columns of $\Phi _{21}$, whereas $\Lambda _{1}$ acts on its rows. This implies that, firstly, if $\Lambda _{1}=\Lambda _{1}(\theta _{\Lambda _{1}})$, respectively, $\Lambda _{2}=\Lambda _{2}(\theta _{\Lambda _{2}})$ are rank-deficient for [almost] all $(\theta _{\Lambda _{1}},\theta _{\Lambda _{2}})$, no row, respectively, column in $\Phi _{21}$ can be fully unrestricted. In other words, no factor from a primary model with rank-deficient loading matrix can covary with all factors of the other primary model. Moreover, in terms of structural requirements for $\Lambda _{1}$, $\Lambda _{2}$, and $\Phi _{21}$, this means that, loosely speaking, if there are linearly dependent columns in $\Lambda _{1}$, respectively, $\Lambda _{2}$, $\Phi _{21}$ should not reflect this dependency in its rows, respectively, columns. More precisely, to obtain an identified composed model, $\Phi _{21}$ should not be decomposable into vectors from $\ker \Lambda _{1}$ in its rows added to vectors from $\ker \Lambda _{2}$ in its columns [cf. Proposition S.8].

In the case of restricted cross-model covariances and rank-deficient loading matrices, neither Item (b) nor Item (c) applies and the condition in Item (a) must be checked directly. This can be achieved by purely algebraic deliberations, such as the calculation of the kernel of $\Lambda _{1}\otimes \Lambda _{2}$ and comparing the resulting equations with those governing the restrictions on $\Phi _{21}$. Alternatively, it can be verified computationally. Indeed, injectivity of $\Lambda _{1}\otimes \Lambda _{2}$ on the subspace of cross-model covariance parameters is obtained if the generating set of $\ker \Lambda _{1}\otimes \Lambda _{2}$ cannot produce vectors in this subspace and vice versa. This is the case if and only if the sum of the dimensions of these spaces equals the dimension of their sum space, which holds because in Theorem 1 all restrictions on $\Phi _{21}$ are assumed to be linear such that the underlying space is a linear subspace. This condition, in turn, can be translated into an equation involving ranks of matrices containing the respective generating sets [see Corollary S.9]. Such an equation can be checked by a modern Computer Algebra System [CAS], such as the free SymPy library (Meurer et al., 2017) for Python. An immediate corollary is that since the rank of $\Lambda _{1}\otimes \Lambda _{2}$ is equal to the product of the ranks of $\Lambda _{1}$ and $\Lambda _{2}$, the number of free covariances in $\Phi _{21}$ cannot exceed this product.

In summary, we obtain the following algorithm to resolve underidentification.

1. Calculate the ranks of both primary models’ loading matrices for [almost] all loading parameter vectors $(\theta _{\Lambda _{1}},\theta _{\Lambda _{2}})$.
2. The product of their ranks then is equal to the maximum number of “free” elements permitted in $\Phi _{21}$.
3. Restrict the remaining number of covariances in $\Phi _{21}$ in a way that leads to identification of the composed model, which can be checked, for example, by verifying Eq. (S.38) algorithmically as demonstrated in the Python code provided as Supplementary Material to this article.

To give an illustration, we tend to our introductory example again. By considering Eq. (7), we find that ${{\,\textrm{rank}\,}}\Lambda _{X}={{\,\textrm{rank}\,}}\Lambda _{Y}=3$ for all $(\gamma _{X2},\gamma _{X3},\gamma _{Y2},\gamma _{Y3})$; that is, both loading matrices are rank-deficient. Note that this still holds if there were more than two items per specific factor with additional loadings as long as the models are Schmid–Leiman solutions to hierarchical models (Yung et al., 1999).

As explicated above, this implies that, firstly, there can be neither fully unrestricted rows nor columns in $\Phi _{YX}$. Secondly, the maximum number of free parameters permitted in $\Phi _{YX}$ is ${{\,\textrm{rank}\,}}\Lambda _{X}\cdot {{\,\textrm{rank}\,}}\Lambda _{Y}=3\cdot 3=9$. Inspecting $\Phi _{YX}$ as defined in Eq. (9), it follows from both preceding deliberations that the model in Fig. 4 is not identified: Both the last row and last column are saturated, and there are $10>9$ free covariances in $\Phi _{YX}$.

To identify the model without changing the loading structure, we set all but the covariances on the diagonal in $\Phi _{YX}$ to zero. Then only $G_{X}$ and $G_{Y}$ as well as every *k*th specific factor $S_{Xk}$ and $S_{Yk}$ from each model are allowed to have nonzero covariance, such that we obtain the altered (now diagonal) cross-model factor covariance matrix

10 $$\begin{aligned} \tilde{\Phi }_{YX}{:}{=}\begin{pmatrix} \sigma _{S_{X1}S_{Y1}} &{} 0 &{} 0 &{} 0\\ 0 &{} \sigma _{S_{X2}S_{Y2}} &{} 0 &{} 0\\ 0 &{} 0 &{} \sigma _{S_{X3}S_{Y3}} &{} 0\\ 0 &{} 0 &{} 0 &{} \sigma _{G_{X}G_{Y}} \end{pmatrix}. \end{aligned}$$

We use the tilde to indicate that this covariance matrix belongs to a new model that differs from the one with covariance matrix $\Phi _{YX}$. Restricting $\Phi _{YX}$ to obtain $\tilde{\Phi }_{YX}$ corresponds to removing all gray dashed lines from Fig. 4. As mentioned above, this cross-model factor covariance matrix renders the model in Fig. 4 a multiconstruct bifactor model (see Koch et al., 2018) with a loading structure according to the hierarchical *G*-factor model.

It is clear that $\tilde{\Phi }_{YX}$ fulfills the necessary identification conditions given above; that is, there are $4\le 9$ free covariances and no saturated rows nor columns. Sufficiency of the structure in $\tilde{\Phi }_{YX}$ is verified in the aforementioned Python script.

Alternatively, the loading matrices can be modified to have full rank for almost all loading parameter values by fixing the loadings of the first item of the general factor to one and setting the other loadings free (i.e., imposing a $\tau$-*congeneric* loading structure; Steyer 2015). Note that the loadings of the items on the specific factors can be set free only if there are at least three items per factor (Steyer et al., 2015). Identification of the composed model then follows from Item (b) in Theorem 1. For completeness, we remark that a composed model with only two indicators per specific factor but unrestricted loadings can be identified *through* cross-model covariances of the specific factors. However, because the intent is to show how identification of a composed model is achieved with identified primary models we do not discuss this case here.

If a $\tau$-congeneric loading structure is used, identification is achieved by adding parameters to the model. It is a well-known fact that there are CFA models for which the more general version may be identified, whereas the more parsimonious is not Bekker et al. (1994). As a simple example, consider a model with two factors indicated by two items each and unrestricted loadings. Bollen (1989, pp. 244–245) showed that the model is identified only if the factors are correlated, whereas the restricted and therefore more parsimonious model with uncorrelated factors is not. The same problem can arise in testing for measurement invariance. For example, Wu and Estabrook (2016) show that researchers can obtain unidentified models when simply adding measurement invariance constraints to a baseline model for ordered categorical outcomes.

If the loading matrix is specified to have full rank for not all, but almost all parameter values (i.e., yielding a generically identified composed model), there are zero-measured regions of the parameter space where the model is not identified. When parameter values lie close to such a region in an empirical application, then this results in an *empirically unidentified* model (see, e.g., Eid et al., 2017; Grayson & Marsh, 1994; Kenny & Kashy, 1992). This, however, is not the case for models with loading matrices that have full rank simply due to their configuration (i.e., their pattern of zero and nonzero entries). The main advantage of these models is that they allow researchers to restrict loadings at will (e.g., to test for measurement invariance) while specifying any cross-model covariance [cf. Item (b)]. We discuss these models in the next section.

## Reduced Models

In the following, we discuss a specific type of model termed *reduced model* and show that taking identified reduced models as primary models results in an identified composed model. We illustrate our findings with the aid of our running example. In Supplementary Material, we give exact definitions and proofs to the deliberations in this section and further distinguish two types of reduced models to be able to determine in which case the composed model is only generically identified.

Recall that an item of *factor complexity one* is an item that has a nonzero loading for exactly one factor (Reilly, 1995). Furthermore, we say that a factor *is associated with* an item if this item has nonzero loading on this factor. Consider some CFA model that contains a factor associated with one or more items with factor complexity one. These items can be considered reference items of this factor, since their variances are assumed to be fully determined by this factor’s variance and the residual (error) variance. In other words, the psychometric meaning of this factor depends on these reference items.

Additionally, assume that there are items of factor complexity two that load on this factor plus one other factor. Although they cannot be considered reference items for the first factor, variation in these items is solely due to variation in the two factors and the residual terms. This variation, in turn, can be interpreted in relation to the variation in the reference items of the first factor.

Put differently, if we disregard the first factor, then variation in these items is solely due to the second factor they are associated with, plus the residual. Holding the first factor constant thus renders these items reference items for the second factor. By the same logic, we can examine the remaining factors for reference items holding previously considered factors constant until we have checked every factor in the model. In summary, we can identify unique contributions to every factor if it is possible to sequentially find factors with reference items, that is, items that load on one factor and not others, disregarding factors already equipped with such items. This is the case if the loading matrix of a model is structured in a way that there is the hypothetical possibility of sequentially removing factors with items of factor complexity one.

It can happen that no such sequence can be found because the factor complexity of all items is greater than one. Nevertheless, it might be possible that dropping a factor from the model altogether, and thus reducing it, allows for such sequences to exist. For this reason, we deem models for which these sequences exist *reduced models*.

To illustrate, consider the loading matrix of any of the two primary models depicted in Fig. 4 and defined in Eq. (7), say $\Lambda _{X}$. All items have factor complexity two; that is, they have nonzero loadings (except for a null set) on exactly two factors for this primary model. This means that it is not possible to begin any sequence finding reference items for every factor because that would require us to start with at least one item with factor complexity one. Consequently, the primary models as defined above and depicted in Fig. 4 are not reduced models.

**Fig. 5:**
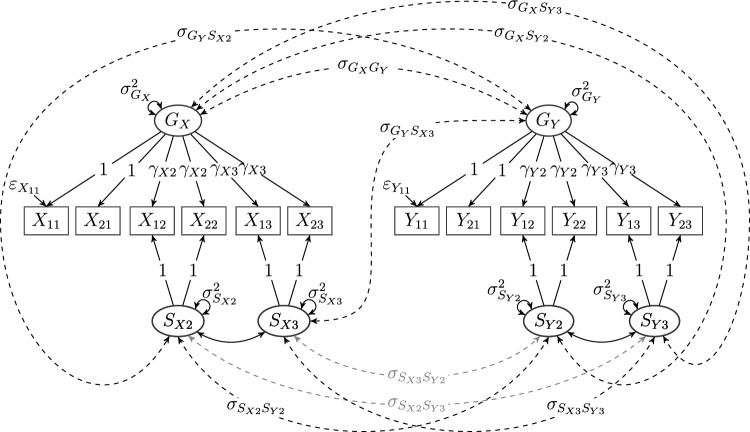
A composed model with two reduced bifactor models as primary models. A reduced bifactor model with items $X_{ji}$, general factor $G_{X}$, and specific factors $S_{X2}$ and $S_{X3}$, composed with another reduced bifactor model with items $Y_{ji}$, general factor $G_{Y}$, and specific factors $S_{Y2}$ and $S_{Y3}$ for $j\in \{1,2\}$ and $i\in \{1,2,3\}$. As the non-reduced version depicted in Fig. 4, items for the first primary (bifactor) model do not load on the second primary (bifactor) model and vice versa. In comparison with the model depicted in Fig. 4, note the additional covariances (black solid lines) that are allowed to be nonzero in the bifactor$(S-1)$ model (Eid et al., 2017). The gray dashed lines between the specific factors *across* the primary models are additional covariances that are allowed to be nonzero while still obtaining an identified composed model.

Nevertheless, dropping any specific factor renders this model a reduced model. Indeed, first observe that this reduces the factor complexity of all items that previously loaded on the dropped specific factor to one. For example, pick the first specific factor to be dropped from the model and redefine the bifactor model as the model with reduced loading matrix

11 $$\begin{aligned} \Lambda ^{\text {R}}_{X}{:}{=}\begin{pmatrix} 0 &{}\quad 0 &{}\quad 1\\ 0 &{}\quad 0 &{}\quad 1\\ 1 &{}\quad 0 &{}\quad \gamma _{X2}\\ 1 &{}\quad 0 &{}\quad \gamma _{X2}\\ 0 &{}\quad 1 &{}\quad \gamma _{X3}\\ 0 &{}\quad 1 &{}\quad \gamma _{X3} \end{pmatrix}. \end{aligned}$$

We use the superscript $\text {R}$ to denote that this loading matrix belongs to a new (reduced) model. It is depicted in Fig. 5 with items $Y_{ji}$, $j\in \{1,2\}$ and $i\in \{1,2,3\}$. Now, the general factor is associated with items $X_{11}$ and $X_{12}$, corresponding to rows 1 and 2 of $\Lambda ^{\text {R}}_{X}$, as items of factor complexity one. Then, a sequence assigning reference items to every factor while disregarding factors already equipped with such items must start with the general factor, since it is the only factor in $\Lambda ^{\text {R}}_{X}$ associated with items of factor complexity one. Disregarding the general factor, the remaining specific factors both only contain items of factor complexity one, which concludes the sequence. In other words, we determine that the primary models in Fig. 5 are reduced models by scanning their loading matrices for items of factor complexity one, disregarding the corresponding factors and repeating this process until all columns have been checked.

In the given example, assume that the (remaining) specific factors are correlated and assume that the general factor is uncorrelated with the specific factors, as depicted in Fig. 5. Then, the model with loading matrix $\Lambda ^{\text {R}}_{X}$ can be denoted a bifactor$(S-1)$ model (Eid et al., 2017), which, in the context of MT-MM research, is known as a correlated traits-correlated methods minus one [CT-C$(M-1)$] model (Eid, 2000). As Geiser et al. (2008) pointed out, there are always as many different possible CT-C$(M-1)$ models as there are methods for any given data. Another example of a reduced model is the bifactor$(S\cdot I-1)$ model (Eid et al., 2017). These models have been shown to possess good psychometric properties and are known for high convergence rates, low number of improper solutions, and a clear psychometric meaning of the latent factors (Eid, 2000; Eid et al., 2017, 2023; Geiser et al., 2008). Which specific factor is dropped from the model, that is, which items loading on the general factor have factor complexity one and thus serve as reference items for the general factor, has implications for the meaning of the general and remaining specific factors (Geiser et al., 2008). However these considerations go beyond the scope of the present article.

In any case, reduced models are not limited to the particular conceptualization of latent variables in the associated measure theoretical framework. The concept of a reduced model is uniquely concerned with the configuration of the loading matrix of the model of interest. To see how reduced models lead to identified composed models, recall from the previous section that $\Lambda _{X}$ is rank-deficient. On the other hand, it is easily verified that $\Lambda ^{\text {R}}_{X}$ has full rank. In fact, every reduced model has this property. Indeed, by removing columns corresponding to factors that are associated with items of factor complexity one, we verify that these columns cannot be represented by the other columns in the loading matrix (because they are nonzero in rows for which the other columns are zero) and since this has to hold for all columns, this property is sufficient for the matrix to have full column rank. From Item (b) of Theorem 1, it then follows immediately that composed models consisting of identified reduced primary models are always identified.

To see how the introductory example can be identified by the use of reduced models, define $\Lambda ^{\text {R}}_{Y}$ similarly to $\Lambda ^{\text {R}}_{X}$—that is, by removing the first column from $\Lambda _{Y}$. The resulting bifactor$(S-1)$ models are still identified (Eid et al., 2017). Then, besides removing the rows and columns of $\Phi _{X}$, $\Phi _{Y}$, and $\Phi _{YX}$ corresponding to the dropped specific factors in the bifactor models, no change is necessary to obtain an identified composed model. The final composed model is depicted in Fig. 5. Moreover, additionally to the covariances between the general factors of one primary model and the specific factors of the other primary model, which were already included in the model depicted in Fig. 4, we can also allow for correlations between the specific factors *across* the primary models, associated with the gray dashed lines in Fig. 5, which results in the reduced but fully unrestricted cross-model covariance matrix

12 $$\begin{aligned} \Phi ^{\text {R}}_{YX}{:}{=}\begin{pmatrix} \sigma _{S_{X2}S_{Y2}} &{}\quad \sigma _{S_{X3}S_{Y2}} &{}\quad \sigma _{G_{X}S_{Y2}}\\ \sigma _{S_{X2}S_{Y3}} &{}\quad \sigma _{S_{X3}S_{Y3}} &{}\quad \sigma _{G_{X}S_{Y3}}\\ \sigma _{S_{X2}G_{Y}} &{}\quad \sigma _{S_{X3}G_{Y}} &{}\quad \sigma _{G_{X}G_{Y}} \end{pmatrix}. \end{aligned}$$

Again, this is because the primary models are reduced models. Thus, the composed model is identified, regardless of the structure of $\Phi ^{\text {R}}_{YX}$, such that allowing for additional covariances to be nonzero in $\Phi ^{\text {R}}_{YX}$ preserves identification.

In summary, working with reduced models simplifies the problem of identification of the composed model to the identification of the primary models. There exists a body of research that is devoted to such a modeling framework and the identification of reduced models, for example, in the context of MT-MM analysis and bifactor applications with a reduced number of specific factors (e.g., Geiser et al., 2008, Koch et al., 2018). Reduced models permit researchers to leave the cross-model factor covariances of the primary models unrestricted, which may be beneficial for the interpretation of the parameters in the composed model (e.g., Eid et al., 2018).

## Discussion

In the present article, we considered the class of composed CFA models. They consist of identified submodels such that, in the complete model, items of one submodel show no cross-loadings on the factors of the other model and error variables are uncorrelated across models. Thus, the submodels only relate to one another via the covariances of their respective common factors.

Although the assumptions of no cross-model loadings and uncorrelated residuals across the primary models may be frequently violated in applied research and may appear strict at first glance,^2^ note that, by the aid of auxiliary factors, the latter can be made without loss of generality and the former can be weakened in some cases. For example, it is straightforward to redefine residual variables as latent variables and incorporate them into the structural part of the model, such that residual covariances across models become cross-model (factor) covariances. For this purpose, an auxiliary factor must be defined with the corresponding item as its unique indicator. For a composed model with nonzero cross-model error covariance matrix $\Psi _{21}$, redefine the primary models’ matrices 13a13b13cfor $i\in \{1,2\}$ and appropriately sized identity matrices $I_i$. Moreover, let13d and define $\hat{\Lambda }_c$, $\hat{\Psi }_c$, and $\hat{\Phi }_c$ accordingly. Then,

14a $$\begin{aligned} \Sigma _i&=\hat{\Lambda }_i\hat{\Phi }_i\hat{\Lambda }_i^T \end{aligned}$$

14b $$\begin{aligned}&=\Lambda _i\Phi _i\Lambda _i^T+\Psi _i \end{aligned}$$

for $i\in \{1,2\}$. Hence, the primary models’ status of identification is unchanged by this procedure.

On the other hand, the equation

15 $$\begin{aligned} \hat{\Sigma }_c=\hat{\Lambda }_c\hat{\Phi }_c\hat{\Lambda }_c^T+\varvec{0} \end{aligned}$$

defines a new composed model without error variables. This way the residual covariances can be considered as cross-model covariances and are therefore subject to the identification conditions given in our theorem. Of course, now the modified counterparts of the loading and cross-model covariance matrices must be used to determine identification. Because the concatenation of the original primary models’ loading matrices with identity matrices renders them rank-deficient, there need to be restrictions on the (augmented) cross-model covariance matrix, now also containing the residual covariances. This implies that the error variables cannot all be correlated across models, even if the (original) loading matrices have full rank. Prospective research could determine which type of cross-model error covariances are possible for which kinds of composed models. In any case, researchers should be aware that allowing for correlated residuals might change the psychometric meaning of some, or even all, factors in the model.

In some cases, cross-model loadings can be dealt with by latent regression on auxiliary factors, which is more involved and beyond the scope of this article. Nevertheless, we sketch it here to show that certain instances of composed models with cross-model loadings are still covered by our theorem. Items for which cross-model loadings are assumed must have their measurement error variables redefined as factors. Former factor loadings are replaced with latent regressions on these new factors within the respective primary model (and thus the regression residuals take the role of the measurement error), again without changing its identification status. In the composed model, cross-model covariances are specified between the redefined item-specific residual variables in one primary model and the factors of the other primary model on which the items are assumed to show cross-loadings. These cross-model covariances, which are identified under the conditions given in our theorem, can be used in a cross-model latent regression analysis. Then, any cross-model loading of interest is given by the regression coefficient of the path connecting the redefined residual variable in the first primary model with the associated factor of the other primary model.

Nevertheless, if cross-model loadings for a majority of items are included, we no longer deem it appropriate to denote the resulting model a composed model. In our view, the two constructs assessed by the two primary models are then no longer sufficiently separable and simply constitute a different, unified type of model. Other modes of identification must be employed, even though a general theorem and proof for the identification of these kinds of models might not be available. In any case, it is important that researchers report on any data-driven changes to their model to avoid engaging in questionable research practices (Flake and Fried, 2020; Crede and Harms, 2019).

We only considered linear constraints on the cross-factor covariances, which make up the overwhelming majority of restrictions on covariances in CFA models.^3^ Nevertheless, the cross-model covariance matrix might be subject to a nonlinear transformation, such as, for example, restricting a covariance to be strictly positive, setting a covariance to be equal some value unequal to zero,^4^ or defining a covariance to be the square of another, just to name a few. However, an extension of our theorem to the nonlinear case is straightforward, since the loading matrices of the primary models act linearly on the (possibly nonlinearly transformed) cross-model covariance matrix. Then, the properties of the nonlinear transformation will be determining the status of identification.

Note that no linearity assumptions are made with respect to the constraints in the primary models. The proof of Theorem S.3 relies on the fact that for identified, and therefore specific, loading matrices $\Lambda _{1}$ and $\Lambda _{2}$, the matrix product $\Lambda _{2}\Phi _{21}\Lambda _{1}^{T}$ is a linear map, regardless of how the loading parameters of the primary models have been mapped into $\Lambda _{1}$ and $\Lambda _{2}$. Thus, we are confident that our theorem applies to the vast majority of composed CFA models encountered in applied research.

Our theorem may also apply to CFA models that initially have not been thought of as composed CFA models, but for which the question of their identification is yet of high practical relevance. One class of examples are multiconstruct LST models (Eid et al., 1994; Schermelleh-Engel et al., 2004). The single-construct LST models they consist of can be considered the primary models, and then, the status of identification for their composition—the multiconstruct LST model—can be determined by our theorem. Even though identification for these types of models has already been shown (Steyer, 1989), they are just one of many conceivable examples of models that are covered by our theorem.

We showed that composed models consisting of reduced primary models must be identified. Working with non-reduced models as primary models, however, can lead to identification issues with the composed model if the loading matrices are rank-deficient as we have shown by means of the introductory example. Note that this strictly follows from algebraic considerations and is not related to the way latent variables are conceptualized. Moreover, it is important to emphasize that non-reduced models, such as the classical bifactor model by Holzinger and Swineford (1939), can still constitute an identified composed model, it just requires additional assumptions. Concretely, researchers must decide if they want to free up parameters and find a suitable full-rank loading structure for the primary models or if there are solid theoretical reasons for why some cross-model covariances should be restricted and if these restrictions necessarily lead to an identified composed model.

We already pointed out the issues of parsimony and empirical underidentification (which we discuss in Supplementary Material) that arise in the former scenario. With this option, measurement invariance assumptions are not testable if they lead to rank-deficiency in the loading matrices of the primary models. As for the latter option, theoretical justification for the restriction of cross-model covariances is given, for example, for interchangeable methods in the LST context (Geiser et al., 2014). Conversely, for structurally different methods, this is not the case. For these kinds of research scenarios, however, there is extensive literature recommending reduced models, such as the CT-C$(M-1)$ model (Nussbeck et al., 2009; Eid et al., 2008).

**Fig. 6:**
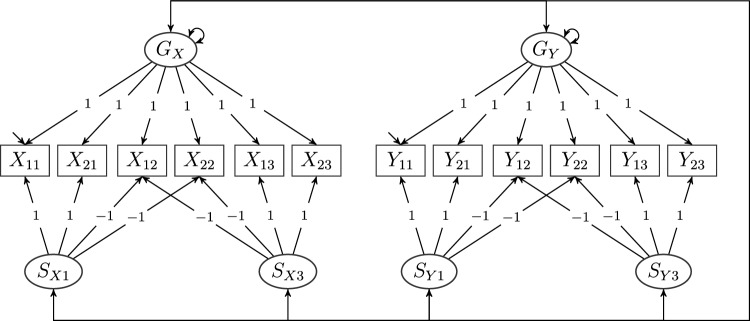
A composed model with two latent means models as primary models. A latent means model with items $X_{ji}$, general factor $G_X$, and specific factors $S_{X1}$ and $S_{X3}$, composed with another latent means model with items $Y_{ji}$, general factor $G_Y$, and specific factors $S_{Y1}$ and $S_{Y3}$, $j\in \{1,2\}$ and $i\in \{1,2,3\}$. All factors are correlated.

It is worth noting that there are models that do not fall under the definition of reduced models as given in the present article, but nevertheless share their favorable properties. Such an example is the latent means model, which could also be used for analyzing designs with structurally different methods (Pohl and Steyer, 2010; Koch et al., 2018). Like the CT-C$(M-1)$ model, it is based on the idea of defining a reduced number of method (or specific) factors. In the latent means model, the trait factor is not parameterized by reference items but as the overall mean of the true score variables pertaining to all structurally different methods. Therefore, the latent means model is not a reduced model. Nevertheless, its loading matrix has full rank under every loading structure, and thus, measurement invariance assumption, as well. The latent means parameterization of our running example is depicted in Fig. 6.

Irrespectively, our results cover a wide range of CFA models which cannot possibly be discussed individually and in full detail. Our theorem allows researchers to easily determine whether a composed CFA model is identified by the necessary and sufficient conditions we provided in this article. In fact, our theorem enables researchers to reduce the problem of identifying the composed model to the problem of identifying the submodels and verifying the conditions stated in Theorem 1. The obstacles for identifying the primary models are precisely those for identifying any arbitrary CFA model: One either must find the specific class this model belongs to and use identification results for this class or explicitly solve for the known parameters algebraically as we discuss in the introduction section. The identification of the primary models hence leads back to the general problem of identifying CFA models that can only be solved for classes of models sharing a certain structure, but not in general.

On the other hand, as the example of a model with two factors and two indicators per factor with free loadings shows, unidentified primary models can be combined in a way that results in a model that is identified *because of* its free cross-model factor covariances (Bollen, 1989). Then, our theorem does not apply and further research is needed to determine in which cases this is possible. However, we take it that, in applied research, models subject to composition are those already employed in practice and extensively studied, such that identification conditions for the primary models are readily available.

Lastly, for complicated primary models that are neither reduced nor equipped with trivially full-rank loading matrices, identification of the composed model can be checked algorithmically. The Python code we supply for the identification of the bifactor models discussed in this article is easily generalized to more sophisticated models. There are no Python skills required beyond defining variables and using the respective libraries’ methods.

We thus supply researchers with a powerful tool to determine their models’ status of identification and lay the mathematical groundwork for discussing a wide range of specific types of composed models that commonly suffer from identification issues.

### Supplementary Information

Below is the link to the electronic supplementary material.

Supplementary file 1 (pdf 237 KB)

## Footnotes

1 In line with the established literature, we assume that the mean structure is identified and do not consider it in the present article (see, e.g., Bollen, 1989).

2 We thank an anonymous reviewer for rightly pointing out this fact which ultimately led to the subsequent addendum.

3 So much so that many authors of papers discussing CFA models even implicitly limit themselves to only considering restrictions in the form of setting some covariances to zero.

4 Note that this restriction can be denoted *affine* linear, but it is not strictly linear in the algebraic sense that is used in the assumptions of our theorem.
