# Supplementary material for: A General Theorem and Proof for the Identification of Composed CFA Models
Source: Psychometrika. 2023 Sep 19;88(4):1334–53. doi: 10.1007/s11336-023-09933-6 (PMC10656336; doi:10.1007/s11336-023-09933-6)
Supplement: Supplementary file 1 — (pdf 237 KB) [file 11336_2023_9933_MOESM1_ESM.pdf]

Supplementary Material

Extended definitions, theorems, and proofs

The Supplementary Material provide a more formal and rigorous treatment of the main article's results. The mathematical proficiency needed to understand the appendices are introductory level linear algebra and calculus, as well as a familiarity with tensor products. For an introduction to the first, we refer to Lang (1987). A comprehensive introduction to matrices can be found in Horn and Johnson (2013). For basic properties of tensor products on vector spaces we used Horn and Johnson (1991) and for a more advanced and abstract algebraic treatment of them we refer to Bourbaki (1989).

Setting

Before introducing notation as well as formalizing the concepts of *primary* and *composed models*, we elucidate some connections between the concept of identification and linear algebra. Recall that a model is identified if, for the model-implied covariance matrix Σ and any arbitrary parameter vectors θ_1 and θ_2, it holds that $\Sigma(\theta_1) \neq \Sigma(\theta_2)$ whenever $\theta_1 \neq \theta_2$ (Bollen, 1989; Jöreskog, 1978). On the one hand, this means that the model parameters can be expressed as a function of the observed variables (Bollen, 1989).

On the other hand this condition equivalently states that a model is identified if its model-implied covariance matrix is an injective map of the parameters. A basic result of linear algebra states that, for linear maps, the injective property can be equivalently expressed in terms of their kernel. Recall that the kernel of a linear map $A : U \to V$, denoted $\ker A \subset U$, is the space of all vectors in U that is mapped to the zero vector in V under A. A basic result of linear algebra then is that a linear map A is injective if and only if its kernel is the trivial subspace consisting only of the zero vector in U, i.e. $\ker A = \{\mathbf{0}\}$. Our proof makes use of the fact that this relation permits to check injectivity of a map via its kernel and therefore determine model identification without the need to explicitly state the function relating the observed variables to the model parameters.

To introduce some notation, consider a CFA model relating (multivariate

real-valued) data $y \in \mathbb{R}^m$ to a (multivariate) random variable η and errors ε, taking values in $\mathbb{R}^n$ and $\mathbb{R}^m$, respectively. With $\alpha \in \mathbb{R}^m$, a constant real vector, and $\Lambda \in \mathbb{R}^{m \times n}$, the loading matrix, the structural equation constituting the model is

$$y = \alpha + \Lambda\eta + \varepsilon. \tag{S.1}$$

As is common practice for CFA models (Bollen, 1989), we assume that η and ε are uncorrelated and that they have zero means as well as finite variance. With the notation

$$\Phi := \mathrm{Var}[\eta], \tag{S.2a}$$

$$\Psi := \mathrm{Var}[\varepsilon], \tag{S.2b}$$

$$\Sigma := \mathrm{Var}[y], \tag{S.2c}$$

it follows that

$$\mathbb{E}[y] = \alpha \tag{S.3}$$

and

$$\Sigma = \Lambda\Phi\Lambda^T + \Psi. \tag{S.4}$$

The covariance matrices Φ and Ψ each contain n and m variances as well as $p \in \{0, \ldots, n(n-1)/2\}$ and $q \in \{0, \ldots, m(m-1)/2\}$ non-zero covariances, respectively. The loading matrix Λ contains some number $r \in \{0, \ldots, mn\}$ of free loadings, while the other $mn - r$ entries in Λ are set to some constant real value such as 0 or 1.

We can collect the free loadings in Λ, variances and covariances of η, and variances and covariances of ε in respective vectors

$$\theta_\Phi \in \mathbb{R}^n_{>0} \times \mathbb{R}^p, \tag{S.5a}$$

$$\theta_\Psi \in \mathbb{R}^m_{>0} \times \mathbb{R}^q, \tag{S.5b}$$

$$\theta_\Lambda \in \mathbb{R}^r. \tag{S.5c}$$

This notation permits to consider the matrices Φ, Ψ, and Λ as functions of their respective entries.

$$\Phi : \mathbb{R}^n_{>0} \times \mathbb{R}^p \to \mathbb{R}^{n \times n}, \tag{S.6a}$$

$$\Psi : \mathbb{R}^m_{>0} \times \mathbb{R}^q \to \mathbb{R}^{m \times m}, \tag{S.6b}$$

$$\Lambda : \qquad \mathbb{R}^r \to \mathbb{R}^{m \times n}. \tag{S.6c}$$

We drop the respective matrices' dependence on parameters whenever it facilitates readability.

Let

$$\mathcal{A} \coloneqq \mathbb{R}^r \times \mathbb{R}^n_{>0} \times \mathbb{R}^p \times \mathbb{R}^m_{>0} \times \mathbb{R}^q, \tag{S.7}$$

the set of all free parameters in the model. We denote $\mathcal{A}$ the CFA model's associated parameter space. This permits to collect θ_Φ, θ_Ψ, and θ_Λ in a vector $\theta \coloneqq (\theta_\Lambda, \theta_\Phi, \theta_\Psi) \in \mathcal{A}$ and consider Σ as a map from the parameter space $\mathcal{A}$ to the space of $m \times m$ matrices, that is,

$$\begin{aligned} \Sigma : \mathcal{A} &\to \mathbb{R}^{m \times m} \\ (\theta_\Lambda, \theta_\Phi, \theta_\Psi) &\mapsto \Lambda(\theta_\Lambda)\Phi(\theta_\Phi)\Lambda(\theta_\Lambda)^T + \Psi(\theta_\Psi). \end{aligned} \tag{S.8}$$

The notation just introduced allows us to reformulate the definition of model identification given above. Following Bekker and ten Berge (1997), we further distinguish a weaker form of global identification, which is *generic* global identification. This notion of identification considers that there might be parameter sets of measure zero where the model is not identified, which is commonly referred to as *theoretical* identification, or simply identification.

If population parameters are close to this set or the characteristics of the data cause parameter estimates to lie in its vicinity, a model may be *empirically* underidentified in a specific application (Kenny & Milan, 2012). This is the case for the example of a two-indicator, two-factor model given in the main body of the text, when

the covariance between factors is close to zero (Bollen, 1989).

Definition S.1. *Consider a CFA model with model-implied covariance matrix* Σ *and parameter space* $\mathcal{A}$*. If* $\Sigma = \Sigma(\theta)$*, as defined in Eq.* (S.8)*, is an injective map [almost] everywhere on* $\mathcal{A}$*, we say that the model is* [generically] globally identified.

By saying that some property holds *everywhere* respectively *almost everywhere* we mean that this property holds for all elements of the parameter space respectively that the set on which this property does not hold is a set of Lebesgue-measure zero. Thus, a model that is globally identified is also generically globally identified, but the converse must not hold.

In the present framework, we assume two CFA models, M_1 and M_2, which we call the *primary models*. The primary models have associated data $y_i \in \mathbb{R}^{m_i}$ as well as latent variables η_i and errors ε_i taking values in $\mathbb{R}^{n_i}$ and $\mathbb{R}^{m_i}$, $i \in \{1, 2\}$, respectively. The loading, latent variable covariance, and error covariance matrices are given by

$$\Lambda_i \in \mathbb{R}^{m_i \times n_i}, \tag{S.9a}$$

$$\Phi_i := \mathrm{Var}[\eta_i] \in \mathbb{R}^{n_i \times n_i}, \tag{S.9b}$$

$$\Psi_i := \mathrm{Var}[\varepsilon_i] \in \mathbb{R}^{m_i \times m_i} \tag{S.9c}$$

for $i \in \{1, 2\}$.

Further denoting the M_i's parameter vectors and parameter spaces as well as mean vectors, data, and model-implied covariance matrices as θ_i, $\mathcal{A}_i$ as well as α_i, y_i, and Σ_i for $i \in \{1, 2\}$, respectively, we get $\mathbb{E}[y_i] = \alpha_i$ and $\Sigma_i(\theta_i) = \Lambda_i \Phi_i \Lambda_i^T + \Psi_i$ for $i \in \{1, 2\}$ [cf. Eqs. (S.3) and (S.4)].

We now combine M_1 and M_2 into a more comprehensive model M_c, which we refer to as the *composed model.* To this end, we concatenate the data, mean vectors,

and latent variables into

$$y_c := (y_1, y_2) \in \mathbb{R}^{m_1} \times \mathbb{R}^{m_2}, \tag{S.10a}$$

$$\alpha_c := (\alpha_1, \alpha_2) \in \mathbb{R}^{m_1} \times \mathbb{R}^{m_2}, \tag{S.10b}$$

$$\eta_c := (\eta_1, \eta_2) \in \mathbb{R}^{n_1} \times \mathbb{R}^{n_2}, \tag{S.10c}$$

$$\varepsilon_c := (\varepsilon_1, \varepsilon_2) \in \mathbb{R}^{m_1} \times \mathbb{R}^{m_2}. \tag{S.10d}$$

As before, assume that the cross-covariance between η_c and ε_c is zero. Furthermore, define the new loading matrix Λ_2 as

$$\Lambda_c := \left(\begin{array}{c|c} \Lambda_1 & \Lambda_{12} \\ \hline \Lambda_{21} & \Lambda_2 \end{array}\right) \in \mathbb{R}^{(m_1+m_2)\times(n_1+n_2)}. \tag{S.11}$$

The structural equation defining the new model M_c is given by

$$y_c = \alpha_c + \Lambda_c \eta_c + \varepsilon_c. \tag{S.12}$$

The latent variable and error covariance matrices are denoted by

$$\Phi_c := \mathrm{Var}[\eta_c] \tag{S.13a}$$

$$= \left(\begin{array}{c|c} \Phi_1 & \Phi_{21}^T \\ \hline \Phi_{21} & \Phi_2 \end{array}\right) \tag{S.13b}$$

and

$$\Psi_c := \mathrm{Var}[\varepsilon_c] \tag{S.14a}$$

$$= \left(\begin{array}{c|c} \Psi_1 & \Psi_{21}^T \\ \hline \Psi_{21} & \Psi_2 \end{array}\right). \tag{S.14b}$$

To distinguish a composed model from any other model with arbitrary block-diagonal structure, we require that the data of both primary models only relate

via the cross-model covariances of the latent variables contained in Φ_{21}. In other words, we assume

$$\Lambda_{21} = \Lambda_{12} = 0 \tag{$\star$}$$

and

$$\Psi_{21} = 0. \tag{$\star\star$}$$

This implies that $\Phi_{21} \in \mathbb{R}^{n_2 \times n_1}$ is the only submatrix introducing new parameters in the composed model. Nevertheless, the covariances contained in Φ_{21} may be subject to certain constraints. To highlight this fact, we collect these covariances in a parameter vector $\theta_{c^*} \in \mathcal{P} \subset \mathbb{R}^{n_2 n_1}$ in a column-major fashion, that is,

$$\theta_{c^*} = \left((\Phi_{21})_{11}, \ldots, (\Phi_{21})_{n_2 1}, \ldots, (\Phi_{21})_{1 n_1}, \ldots, (\Phi_{21})_{n_2 n_1}\right), \tag{S.15}$$

and consider Φ_{21} as a function of these parameters (note that Φ_{21} defined in this way is an injection),

$$\begin{aligned} \Phi_{21} : \mathcal{P} &\to \mathbb{R}^{n_2 \times n_1} \\ \theta_{c^*} &\mapsto \Phi_{21}(\theta_{c^*}). \end{aligned} \tag{S.16}$$

Moreover, we assume that $\mathcal{P}$ is a subspace of $\mathbb{R}^{n_2 n_1}$, which implies that we only allow structure-preserving constraints, such as, for example, zero or proportionality constraints. This also implies that no new parameters are introduced in the constraints on the cross-model covariances.

For M_c, we define the parameter vector $\theta_c := (\theta_1, \theta_2, \theta_{c^*}) \in \mathcal{A}_c$ with $\mathcal{A}_c := \mathcal{A}_1 \times \mathcal{A}_2 \times \mathcal{P}$, that is, a collection of the primary models' parameter vectors as well as θ_{c^*}. To be able to refer to the parameter vectors of M_c belonging to the different matrices individually, we also define $\theta_{\Lambda_c} := (\theta_{\Lambda_1}, \theta_{\Lambda_2})$, $\theta_{\Phi_c} := (\theta_{\Phi_1}, \theta_{\Phi_2}, \theta_{c^*})$, and $\theta_{\Psi_c} := (\theta_{\Psi_1}, \theta_{\Psi_2})$.

With these definitions, we can again consider Σ_c as a function of the model's

parameters θ_c and write

$$\Sigma_c(\theta_c) = \Lambda_c \Phi_c \Lambda_c^T + \Psi_c. \tag{S.17}$$

Theorem and Proof

Theorem S.3 is stated in terms of the Kronecker product of matrices. For two matrices $A \in \mathbb{R}^{p \times q}$ and $B \in \mathbb{R}^{r \times s}$ it is defined as

$$A \otimes B := \begin{pmatrix} a_{11}B & \dots & a_{1q}B \\ \vdots & \ddots & \vdots \\ a_{p1}B & \dots & a_{pq}B \end{pmatrix} \in \mathbb{R}^{pr \times qs}. \tag{S.18}$$

For a concise introduction to the Kronecker product, see Horn and Johnson (1991).

However, the Kronecker product of matrices acting on vector spaces can be considered a special case of the tensor product, a more abstract operation defined on modules. This abstraction allows for the use of more powerful theorems to derive properties of the Kronecker product. For details we refer to Bourbaki (1989).

As a preliminary to the subsequent considerations, we extend the notation of the Kronecker product to vector spaces in the following way.

Proposition S.2. *Let $U \subset \mathbb{R}^p$ and $V \subset \mathbb{R}^q$ be vector spaces. Then*

$$U \otimes V := \{u \otimes v \in \mathbb{R}^{pq} : u \in U, v \in V\} \tag{S.19}$$

is a vector space.

Proof. Omitted, see Bourbaki (1989) for details. ■

The preceding remarks allow us to state the central result of the present article as follows.

Theorem S.3. *Consider a composed model as previously defined and let the primary models be [generically] globally identified. Then the composed model is [generically] globally identified if and only if*

$$\ker\left(\Lambda_1(\theta_{\Lambda_1}) \otimes \Lambda_2(\theta_{\Lambda_2})\right) \cap \mathcal{P} = \{\mathbf{0}\} \tag{S.20}$$

for [almost] all $(\theta_{\Lambda_1}, \theta_{\Lambda_2})$.

Proof. We first show that the composed model being generically globally identified implies that the primary models are generically globally identified as well. Then, assuming generic global identification of the primary models, we show that the composed model's generic global identification is equivalent to Eq. (S.20). The property of the composed model being globally identified everywhere is proved analogously.

For all subsequent remarks it is crucial to consider the following block structure of $\Sigma_c(\theta_c)$ implied by Eqs. (S.13) and (S.14) as well as assumptions ($\star$) and ($\star\star$):

$$\Sigma_c(\theta_c) = \left(\begin{array}{c|c} \Lambda_1\Phi_1\Lambda_1^T + \Psi_1 & \Lambda_1\Phi_{21}^T\Lambda_2^T \\ \hline \Lambda_2\Phi_{21}\Lambda_1^T & \Lambda_2\Phi_2\Lambda_2^T + \Psi_2 \end{array}\right) \tag{S.21a}$$

$$= \left(\begin{array}{c|c} \Sigma_1(\theta_1) & \Lambda_1\Phi_{21}^T\Lambda_2^T \\ \hline \Lambda_2\Phi_{21}\Lambda_1^T & \Sigma_2(\theta_2) \end{array}\right). \tag{S.21b}$$

Let the composed model be generically globally identified and let $\theta_i = (\theta_{\Lambda_i}, \theta_{\Phi_i}, \theta_{\Psi_i}) \in \mathcal{A}_i$ as well as $\tilde{\theta}_i = (\tilde{\theta}_{\Lambda_i}, \tilde{\theta}_{\Phi_i}, \tilde{\theta}_{\Psi_i}) \in \mathcal{A}_i$ such that $\Sigma_i(\theta_i) = \Sigma_i(\tilde{\theta}_i)$ for $i \in \{1, 2\}$. For notational brevity, we write $\tilde{\Lambda}_i := \Lambda_i(\tilde{\theta}_{\Lambda_i})$, $\tilde{\Phi}_i := \Phi_i(\tilde{\theta}_{\Phi_i})$, and $\tilde{\Psi}_i := \Psi_i(\tilde{\theta}_{\Psi_i})$.

Now pick $\theta_{c^*}, \tilde{\theta}_{c^*} \in \mathcal{P}$ such that $\Lambda_2\Phi_{21}(\theta_{c^*})\Lambda_1^T = \tilde{\Lambda}_2\Phi_{21}(\tilde{\theta}_{c^*})\tilde{\Lambda}_1^T$. Note that such $\theta_{c^*}, \tilde{\theta}_{c^*}$ exist since $\mathcal{P}$ is a subspace and we can pick $\theta_{c^*}, \tilde{\theta}_{c^*} := \mathbf{0} \in \mathcal{P}$. Then $\Phi_{21}(\mathbf{0}) = \mathbf{0}$ and therefore $\Lambda_2\Phi_{21}(\mathbf{0})\Lambda_1^T = \tilde{\Lambda}_2\Phi_{21}(\mathbf{0})\tilde{\Lambda}_1^T$.

But then we have $\theta_c := (\theta_1, \theta_2, \theta_{c^*})$ as well as $\tilde{\theta}_c := (\tilde{\theta}_1, \tilde{\theta}_2, \tilde{\theta}_{c^*})$ such that, by Eq. (S.21b), $\Sigma_c(\theta_c) = \Sigma_c(\tilde{\theta}_c)$. By assumption, it follows that $\theta_c = \tilde{\theta}_c$ for almost all such $\theta_c, \tilde{\theta}_c$ and therefore $\theta_i = \tilde{\theta}_i$ for $i \in \{1, 2\}$ almost everywhere. Thus, the primary models are generically globally identified.

Conversely, assume that the primary models are generically globally identified and pick θ_c and $\tilde{\theta}_c$ such that $\Sigma_c(\theta_c) = \Sigma_c(\tilde{\theta}_c)$. In the following, we use the same notation as before as well as $\tilde{\Phi}_{21} := \Phi_{21}(\tilde{\theta}_{c^*})$.

Then, comparing the diagonal submatrices of $\Sigma_c(\theta_c)$ and $\Sigma_c(\tilde{\theta}_c)$, we find that $\Sigma_i(\theta_i) = \Sigma_i(\tilde{\theta}_i)$ for $i \in \{1, 2\}$. By assumption, it follows that $\Lambda_i = \tilde{\Lambda}_i$, $\Phi_i = \tilde{\Phi}_i$, and

$\Psi_i = \tilde{\Psi}_i$ and therefore $\theta_i = \tilde{\theta}_i$ for $i \in \{1, 2\}$ almost everywhere. Hence, it only remains to be shown that Eq. (S.20) holds if and only if $\theta_{c^*} = \tilde{\theta}_{c^*}$ or, equivalently, $\Phi_{21} = \tilde{\Phi}_{21}$ almost everywhere.

To that end define the linear map

$$\Omega(\Phi_{21}) := \Lambda_2 \Phi_{21} \Lambda_1^T. \tag{S.22}$$

Comparison of the off-diagonal block matrices of $\Sigma_c(\theta_c)$ and $\Sigma_c(\tilde{\theta}_c)$ provides

$$\begin{aligned}
\Omega(\Phi_{21}) &= \Lambda_2 \Phi_{21} \Lambda_1^T && \text{(S.23a)}\\
&= \tilde{\Lambda}_2 \tilde{\Phi}_{21} \tilde{\Lambda}_1^T && \text{(S.23b)}\\
&= \Lambda_2 \tilde{\Phi}_{21} \Lambda_1^T && \text{(S.23c)}\\
&= \Omega(\tilde{\Phi}_{21}). && \text{(S.23d)}
\end{aligned}$$

With the isomorphism $\text{vec}_{r,s}(a_1|\ldots|a_s) = (a_1, \ldots, a_s)^T \in \mathbb{R}^{rs}$ stacking the columns of a matrix into a vector, it follows that (Lemma 4.3.1 in Horn & Johnson, 1991) $(\Lambda_1 \otimes \Lambda_2)\,\text{vec}(\Phi_{21}) = \text{vec}(\Omega(\Phi_{21}))$ and therefore $\Omega = \text{vec}^{-1} \circ (\Lambda_1 \otimes \Lambda_2) \circ \text{vec}$. Further note that $\text{vec}(\Phi_{21}(\theta_{c^*})) = \theta_{c^*} \in \mathcal{P}$. Consequently,

$$\begin{aligned}
\text{vec}(\Omega(\Phi_{21})) &= (\Lambda_1 \otimes \Lambda_2)\,\text{vec}(\Phi_{21}) && \text{(S.24a)}\\
&= (\Lambda_1 \otimes \Lambda_2)\,\text{vec}(\Phi_{21}(\theta_{c^*})) && \text{(S.24b)}\\
&= (\Lambda_1 \otimes \Lambda_2)\theta_{c^*}. && \text{(S.24c)}
\end{aligned}$$

By Eq. (S.23d), we have that $(\Lambda_1 \otimes \Lambda_2)\theta_{c^*} = (\Lambda_1 \otimes \Lambda_2)\tilde{\theta}_{c^*}$ if and only if $(\Lambda_1 \otimes \Lambda_2)(\theta_{c^*} - \tilde{\theta}_{c^*}) = 0$ or, equivalently, $\theta_{c^*} - \tilde{\theta}_{c^*} \in \ker(\Lambda_1 \otimes \Lambda_2)$. Thus, we can conclude that $\theta_{c^*} = \tilde{\theta}_{c^*}$, with $\theta_{c^*}, \tilde{\theta}_{c^*} \in \mathcal{P}$, if and only if $\Lambda_1 \otimes \Lambda_2 = \Lambda_1(\theta_{\Lambda_1}) \otimes \Lambda_2(\theta_{\Lambda_2})$ is injective on $\mathcal{P}$. This condition, however, is equivalent to Eq. (S.20).

In summary, if the composed model is identified for almost all parameters, that is, we can conclude $\theta_{c^*} = \tilde{\theta}_{c^*}$ for almost all parameters, then Eq. (S.20) must hold for almost all $(\theta_{\Lambda_1}, \theta_{\Lambda_2})$. Conversely, if Eq. (S.20) holds for almost all $(\theta_{\Lambda_1}, \theta_{\Lambda_2})$, then

$\theta_{c^*} = \tilde{\theta}_{c^*}$ for almost all parameters if injectivity holds for the primary models' submatrices for almost all parameters.

However, even if Eq. (S.20) holds for all parameters, if the primary models are only generically globally identified, there are still measure zero sets for which the primary models and thus the composed model is not identified. Therefore, for the composed model to be globally identified everywhere, in addition to Eq. (S.20) being true for all parameter values, the primary models must be globally identified everywhere as well. ■

Remark S.4. *If the primary models are globally identified, the composed model is generically globally identified if and only if Eq.* (S.20) *holds almost everywhere.*

Proof. The primary models being globally identified implies that they are generically globally identified as well. Then by Theorem S.3, for the composed model to be generically globally identified, it suffices that Eq. (S.20) holds almost everywhere. ■

Stating the identification conditions in Theorem S.3 in terms of a kernel is rather technical, but it permits to directly relate rank information of the primary model's loading matrices to the composed model's status of identification. This relation will be proved in the subsequent Corollaries.

Conceptually, however, Eq. (S.20) can best be understood in terms of the final remarks of the proof. With the isomorphism vec, $\Lambda_1 \otimes \Lambda_2$ can be seen as a linear map acting on matrices from the space $\mathcal{P}$. Considering the relation between injectivity and the kernel of a map, Eq. (S.20) just expresses the condition that Φ_{21} can be reconstructed from the product $\Lambda_2 \Phi_{21} \Lambda_1^T$.

Corollary S.5. *In the setting of Theorem S.3, suppose that the primary models are [generically] globally identified and that Λ_1 as well as Λ_2 are of full column rank for [almost] all parameters. Then the composed model is [generically] globally identified.*

Proof. If both Λ_1 and Λ_2 are of full column rank for [almost] all parameters, then $\ker \Lambda_1 = \ker \Lambda_2 = \{\mathbf{0}\}$ and therefore Eq. (S.20) holds for [almost] all parameters. By Theorem S.3, if the primary models are [generically] globally identified, then the composed model is [generically] globally identified as well. ■

Corollary S.6. *In the setting of Theorem S.3, suppose that the primary models are [generically] globally identified and further suppose that* Φ_{21} *is saturated, that is,*

$$\mathcal{P} = \mathbb{R}^{n_2 n_1}. \tag{S.25}$$

Then the composed model is [generically] globally identified if and only if both Λ_1 *and* Λ_2 *are of full column rank for [almost] all parameters.*

Proof. For sufficiency, see Corollary S.5. For necessity, we use the fact that $\operatorname{rank} \Lambda_1 \otimes \Lambda_2 = \operatorname{rank} \Lambda_1 \operatorname{rank} \Lambda_2$ (Theorem 4.2.15 in Horn & Johnson, 1991). Thus if any one of Λ_1 and Λ_2 has insufficient column rank for a parameter set of positive measure [respectively for a non-empty set of at least measure zero], then so does $\Lambda_1 \otimes \Lambda_2$. But then $\Lambda_1 \otimes \Lambda_2$ has a nontrivial kernel and therefore

$$\begin{aligned} \{\mathbf{0}\} &\neq \ker(\Lambda_1 \otimes \Lambda_2) && \text{(S.26a)} \\ &= \ker(\Lambda_1 \otimes \Lambda_2) \cap \mathbb{R}^{n_2 n_1} && \text{(S.26b)} \\ &= \ker(\Lambda_1 \otimes \Lambda_2) \cap \mathcal{P}. && \text{(S.26c)} \end{aligned}$$

Therefore, by Theorem S.3, the composed model is not [generically] globally identified. ■

In the case that any one or both of Λ_1 and Λ_2 have nontrivial kernels, in order to pick a suitable $\mathcal{P}$ we must gain insight into the structure of $\ker(\Lambda_1 \otimes \Lambda_2)$. For this purpose, Proposition S.8 gives a decomposition into separate spaces involving the kernels of Λ_1 and Λ_2.

In order to proof Proposition S.8, however, we need the following lemma.

Lemma S.7. *Let* $A \in \mathbb{R}^{p \times q}$ *and* $B \in \mathbb{R}^{r \times s}$ *be matrices. Then*

$$\ker(A \otimes B) = \mathbb{R}^{p \times q} \otimes (\ker B) + (\ker A) \otimes \mathbb{R}^{r \times s}. \tag{S.27}$$

Proof. In Proposition 6, Chapter II.3 in Bourbaki (1989) this is proved for homomorphisms on flat modules. Since matrices are vector space homomorphisms and

vector spaces are free modules and therefore flat (Bourbaki, 1989), the claim follows. Regardless, we give an additional proof in terms of the Kronecker product that does not require abstract algebra.

Let $V := \mathbb{R}^q$, $W := \mathbb{R}^p$, $V' := \mathbb{R}^r$, and $W' := \mathbb{R}^s$ and define

$$K := V \otimes (\ker B) + (\ker A) \otimes V'. \tag{S.28}$$

Furthermore, pick U as a complement to $\ker A$ and U' as a complement to $\ker B$. We now show that

$$V \otimes V' = (U \otimes U') \oplus K. \tag{S.29}$$

Take $k \in V \otimes V'$. Since $V = U \oplus \ker A$ and $V' = U' \oplus \ker B$, there exist $x \in U$ and $p \in \ker A$ as well as $y \in U'$ and $q \in \ker B$ such that

$$k = (x + p) \otimes (y + q) \tag{S.30a}$$

$$= x \otimes y + (x + p) \otimes q + p \otimes y \tag{S.30b}$$

by the bilinearity of the Kronecker product (4.2.7 and 4.2.8 in Horn & Johnson, 1991). Since $x \otimes y \in U \otimes U'$ and $(x + p) \otimes q + p \otimes y \in K$, it follows that

$$V \otimes V' = (U \otimes U') + K. \tag{S.31}$$

If $k \in K$, then there exist $v \in V$ and $q \in \ker B$ as well as $v' \in V'$ and $p \in \ker A$ such that $k = v \otimes q + p \otimes v'$. Then, using Lemma 4.2.10 by Horn and Johnson (1991),

$$(A \otimes B)k = (A \otimes B)(v \otimes q + p \otimes v') \tag{S.32a}$$

$$= (A \otimes B)(v \otimes q) + (A \otimes B)(p \otimes v') \tag{S.32b}$$

$$= (Av) \otimes (Bq) + (Ap) \otimes (Bv') \tag{S.32c}$$

$$= (Av) \otimes \mathbf{0} + \mathbf{0} \otimes (Bv') \tag{S.32d}$$

$$= \mathbf{0}. \tag{S.32e}$$

On the other hand, if $u \in U \otimes U'$, then there exist $x \in U$ and $y \in U'$ such that $u = x \otimes y$. Moreover, $Ax = \mathbf{0}$ if and only if $x = \mathbf{0}$ and, similarly, $By = \mathbf{0}$ if and only if $y = \mathbf{0}$ by construction of U and U'. Therefore,

$$(A \otimes B)u = (A \otimes B)(x \otimes y) \tag{S.33a}$$
$$= (Ax) \otimes (By) \tag{S.33b}$$
$$= \mathbf{0} \tag{S.33c}$$

if and only if either $x = \mathbf{0}$ or $y = \mathbf{0}$ (p. 244 in Horn & Johnson, 1991) and thus $u = \mathbf{0}$. It follows that $(U \otimes U') \cap K = \mathbf{0}$, which proves the claim.

Moreover, the last remark shows that $A \otimes B$ is split, vanishing only on K. It follows that $K = \ker(A \otimes B)$, which proves the lemma. ■

Proposition S.8. *As in Eq.* (S.22)*, define* $\Omega(\Phi_{21}) := \Lambda_2 \Phi_{21} \Lambda_1^T$*. Then*

$$\ker \Omega = (\ker \Lambda_1)^T \otimes \mathbb{R}^{n_2} + (\mathbb{R}^{n_1})^T \otimes (\ker \Lambda_2). \tag{S.34}$$

Proof. Lemma S.7 implies $\ker(\Lambda_1 \otimes \Lambda_2) = (\ker \Lambda_1) \otimes \mathbb{R}^{n_2} + \mathbb{R}^{n_1} \otimes (\ker \Lambda_2)$ and therefore

$$\ker \Omega = \ker\left(\mathrm{vec}^{-1} \circ (\Lambda_1 \otimes \Lambda_2) \circ \mathrm{vec}\right) \tag{S.35a}$$
$$= \mathrm{vec}^{-1}\left((\Lambda_1 \otimes \Lambda_2)^{-1}\left(\ker \mathrm{vec}^{-1}\right)\right) \tag{S.35b}$$
$$= \mathrm{vec}^{-1}\left(\ker(\Lambda_1 \otimes \Lambda_2)\right) \tag{S.35c}$$
$$= \mathrm{vec}^{-1}\left(\ker \Lambda_1 \otimes \mathbb{R}^{n_2} + \mathbb{R}^{n_1} \otimes \ker \Lambda_2\right) \tag{S.35d}$$
$$= (\ker \Lambda_1)^T \otimes \mathbb{R}^{n_2} + (\mathbb{R}^{n_1})^T \otimes \ker \Lambda_2 \tag{S.35e}$$

as claimed. ■

In the previous case and if $\mathcal{P}$ is chosen such that $\mathcal{P} \subsetneq \mathbb{R}^{n_2 n_1}$, Eq. (S.20) can be checked by calculating the dimensions of the subspaces involved. For this purpose, pick a generating set $G = \{g_1, \ldots, g_p\}$ for $\ker(\Lambda_1 \otimes \Lambda_2)$ and a generating set $D = \{d_1, \ldots, d_q\}$

for $\mathcal{P}$ and assemble both G and D column-wise in respective matrices

$$\Gamma \coloneqq \left(g_1 \middle| \dots \middle| g_p \right) \tag{S.36}$$

and

$$\Delta \coloneqq \left(d_1 \middle| \dots \middle| d_p \right). \tag{S.37}$$

Corollary S.9. *The composed model is [generically] globally identified if and only if the primary models are [generically] globally identified and*

$$\operatorname{rank}\left(\Delta \middle| \Gamma \right) = \operatorname{rank}\Delta + \operatorname{rank}\Gamma \tag{S.38a}$$

$$= \operatorname{rank}\Delta + n_1 n_2 - \operatorname{rank}\Lambda_1 \operatorname{rank}\Lambda_2 \tag{S.38b}$$

holds [almost] everywhere.

Proof. It holds that $\operatorname{rank}\Gamma = \dim\left(\ker(\Lambda_1 \otimes \Lambda_2)\right)$. Thus, by the definition of a rank, Eq. (S.38a) holding almost everywhere, respectively everywhere, is equivalent to

$$\dim(\mathcal{P} + \ker(\Lambda_1 \otimes \Lambda_2)) = \dim \mathcal{P} + \dim \ker(\Lambda_1 \otimes \Lambda_2) \tag{S.39}$$

if and only if $\dim(\ker(\Lambda_1 \otimes \Lambda_2) \cap \mathcal{P}) = 0$ almost everywhere, respectively everywhere. This, in turn, is equivalent to Eq. (S.20) holding almost everywhere, respectively everywhere.

For Eq. (S.38b), we again use the fact that $\operatorname{rank}\Lambda_1 \otimes \Lambda_2 = \operatorname{rank}\Lambda_1 \operatorname{rank}\Lambda_2$. (Theorem 4.2.15 in Horn & Johnson, 1991). Hence,

$$n_1 n_2 = \dim \mathbb{R}^{n_1 n_2} \tag{S.40a}$$

$$= \dim \operatorname{Im}(\Lambda_1 \otimes \Lambda_2) + \dim \ker(\Lambda_1 \otimes \Lambda_2) \tag{S.40b}$$

$$= \operatorname{rank}\Lambda_1 \otimes \Lambda_2 + \operatorname{rank}\Gamma \tag{S.40c}$$

$$= \operatorname{rank}\Lambda_1 \operatorname{rank}\Lambda_2 + \operatorname{rank}\Gamma, \tag{S.40d}$$

which proves Eq. (S.38b) and thus the corollary. ■

Reduced models

In the following, we formalize the notion of reduced models given in the article. Recall that we call a model reduced if its loading matrix is structured in a way that there is the hypothetical possibility of sequentially removing factors with items of factor complexity one. To be more explicit, given a loading matrix $\Lambda \in \mathbb{R}^{m\times n}$, we say that an item is of factor complexity one *with respect to* Λ if it has nonzero loading for exactly one factor in Λ. To arrive at a formal definition, we show how such a sequence can be construed for illustrative example from the main article.

The starting point is the redefined loading matrix of the first primary model depicted in Fig. 5 in the main article,

$$\tilde{\Lambda}_X := \begin{pmatrix} 0 & 0 & 1 \\ 0 & 0 & 1 \\ 1 & 0 & \gamma_2 \\ 1 & 0 & \gamma_2 \\ 0 & 1 & \gamma_3 \\ 0 & 1 & \gamma_3 \end{pmatrix}. \tag{S.41}$$

To define the sequence, take the column indices as its members. There is three factors and thus there is three sequence members k_1, k_2, and k_3. Since the general factor corresponds to the third column in $\tilde{\Lambda}_X$, we need to set $k_1 := 3$.

The general factor is associated with an item of factor complexity one and is therefore equipped with a reference item and we exclude it from the following choices of sequence members. The definition requires iterating through the remaining columns until there are no factors left to be assigned with reference items. To implement this idea, we define a sequence of submatrices $\tilde{\Lambda}_X^{(k)}$ of $\tilde{\Lambda}_X$, $k \in \{1, 2, 3\}$, alongside the sequence of column indices. This sequence starts with the full matrix from which we choose the general factor to be assigned with reference items, that is, $\tilde{\Lambda}_X^{(1)} = \tilde{\Lambda}_X$.

Excluding the general factor then means defining the next submatrix in the

sequence as missing the column corresponding to the general factor. In the present example, it follows that the next sequence member $\tilde{\Lambda}_X^{(2)}$ should only contain the first and second column of $\tilde{\Lambda}_X$, which we express by the notation

$$\tilde{\Lambda}_X^{(2)} := \tilde{\Lambda}_X|_{\{1,2\}} = \begin{pmatrix} 0 & 0 \\ 0 & 0 \\ 1 & 0 \\ 1 & 0 \\ 0 & 1 \\ 0 & 1 \end{pmatrix}. \tag{S.42}$$

Here, $\tilde{\Lambda}_X|_{\{1,2\}}$ designates the submatrix of $\tilde{\Lambda}_X$ that consists of only the columns with column indices 1 and 2.

Possible choices for the next member of the sequence of column indices are $k_2, k_3 \in \{1, 2\}$. Because both factors contain items of factor complexity one, we are free to chose the column index corresponding to any of the two as the next sequence member. Pick $k_2 := 1$. Then $k_3 = 2$ and

$$\Lambda_X^{(3)} := \tilde{\Lambda}_X|_{\{2\}} = \begin{pmatrix} 0 \\ 0 \\ 0 \\ 0 \\ 1 \\ 1 \end{pmatrix}, \tag{S.43}$$

even though we might have well picked $k_3 = 1$ and hence $\Lambda_X^{(3)} := \tilde{\Lambda}_X|_{\{1\}}$.

In short, to determine that the model with loading matrix $\tilde{\Lambda}_X$ is a reduced model, we have found a sequence of column indices of length three assigning reference items to each factor and disregarding them in the next step by considering a smaller submatrix. The following definition is a generalization of this iterative process that takes the distinction of generically and every identified composed models into account.

Definition S.10 (Conditionally and unconditionally reduced models)**.** *Let* $\Lambda = (\boldsymbol{\lambda}_k)_{k\in\{1,\dots,n\}} \in \mathbb{R}^{m\times n}$ *be the loading matrix of a CFA model with* $\boldsymbol{\lambda}_k \in \mathbb{R}^{m\times 1}$ *for* $k \in \{1,\dots,n\}$*, the columns of* Λ*. Denote the submatrix of* Λ *containing only the columns with column indices in the set* $K \subseteq \{1,\dots,n\}$ *as* $\Lambda|_K := (\boldsymbol{\lambda}_k)_{k\in K} \in \mathbb{R}^{m\times|K|}$*.*

If there exists a sequence of n *distinct column indices* $k_1,\dots,k_n \in \{1,\dots,n\}$*, and a sequence of submatrices* $\Lambda^{(j)} := \Lambda|_{\{k_j,\dots,k_n\}} \in \mathbb{R}^{m\times(n-j+1)}$*,* $j \in \{1,\dots,n\}$*, such that the factor corresponding to the* k_j*th column is associated with at least one item of factor complexity one with respect to* $\Lambda^{(j)}$ *for* $j \in \{1,\dots,n\}$*, we say that the CFA model is* reduced*.*

If there exists such a sequence and if the loadings of all items of factor complexity one w.r.t. $\Lambda^{(j)}$ *that load on the factor corresponding to the* k_j*th column for* $j \in \{1,\dots,n\}$ *are parameters, then we say that the model is* conditionally reduced*.*

If there exists such a sequence and if there exists an item of factor complexity one w.r.t. $\Lambda^{(j)}$ *that has unit loading on the factor corresponding to the* k_j*th column for* $j \in \{1,\dots,n\}$*, then we say that the model is* unconditionally reduced*.*

Definition S.10 is fulfilled if the loading matrix of a model is structured in a way that there is the hypothetical possibility of sequentially removing factors with items of factor complexity one. As we showed for the introductory example, the definition is easily verified by scanning a loading matrix for items of factor complexity one, disregarding the corresponding factors and repeating this process until all columns have been checked.

We return to the running example from the main body of the text to illustrate the distinction between conditionally and unconditionally reduced models. Consider Λ_X as defined in Eq. (7). As was shown, this model is not reduced and Λ_X is rank-deficient.

Although imposing a τ-congeneric loading structure (i.e., setting the loadings of every first item on every factor to unity and freeing up the other loadings) results in a full-rank loading matrix, this only holds for almost all parameter values. This is because all loadings that have been freed up in the τ-congeneric loading structure could still potentially assume the value one. Then an essentially τ-equivalent loading

structure holds and the loading matrix is rank-deficient again. As discussed before, this means that there can be data for which the model is empirically underidentified.

This is in contrast to $\tilde{\Lambda}_X$ as defined in Eq. (11) in the main article. Here, even if we assume a τ-congeneric loading structure, then the new model would still be an unconditionally reduced model and the new loading matrix would still have full rank for all parameter values. The following lemma specifies this property for both types of reduced models as defined in Definition S.10.

Lemma S.11. *Let $\Lambda \in \mathbb{R}^{m\times n}$ be the loading matrix of a reduced CFA model. Then Λ has full rank for almost all parameter values. Specifically,*

(a) if the model is conditionally reduced, then there is a set of parameter values for which Λ does not have full rank

and

(b) if the model is unconditionally reduced, Λ has full rank for all parameter values.

Proof. We prove this by induction over n, the number of columns in Λ. For $n = 1$, Definition S.10 states that there is a column index $k_1 = 1$ such that $\Lambda^{(1)} := \Lambda|_{\{k_1\}} = \Lambda$ possesses at least one item of factor complexity one. For $n = 1$ this implies that there is at least one nonzero loading in the single-column matrix Λ. If all nonzero loadings are parameters, then if all parameters are equal to zero, Λ has rank zero. If there is a unit loading, then $\operatorname{rank}\Lambda = 1$ always. This proves the base case.

Now let $n \in \mathbb{N}$ and $\Lambda \in \mathbb{R}^{m\times(n+1)}$. By assumption, $\Lambda \in \mathbb{R}^{m\times(n+1)}$ is the loading matrix of a reduced model. Thus, by the definition of a reduced model, there exists a sequence of $n+1$ distinct column indices $k_1, \ldots, k_{n+1} \in \{1, \ldots, n+1\}$, and a sequence of submatrices $\Lambda^{(j)} := \Lambda|_{\{k_j,\ldots,k_{n+1}\}}$, $j \in \{1, \ldots, n+1\}$, such that the factor corresponding to the k_jth column possesses at least one item of factor complexity one w.r.t. $\Lambda^{(j)}$ for $j \in \{1, \ldots, n+1\}$.

In particular, the factor corresponding to the k_1th column possesses at least one item of factor complexity one w.r.t. $\Lambda^{(1)} = \Lambda|_{\{k_1,\ldots,k_{n+1}\}} = \Lambda$. By definition, any such item has nonzero loading on the factor indicated by the k_1th column $\boldsymbol{\lambda}_{k_1}$ and zero

loadings on the remaining factors, that is, the row it corresponds to has zero entries in all but $\boldsymbol{\lambda}_{k_1}$. If the loadings of all such items are parameters, then $\boldsymbol{\lambda}_{k_1}$ is linearly independent of all other columns in Λ for at least almost all parameter values. On the other hand, if there is one item with unit loading, then $\boldsymbol{\lambda}_{k_1}$ is linearly independent of all other columns in Λ for all parameter values.

Remove the k_1th column by defining $\tilde{\Lambda} := \Lambda|_{\{k_2,\ldots,k_{n+1}\}} \in \mathbb{R}^{m \times n}$. Then the sequence

$$l_m := \begin{cases} k_{m+1} \in \{1, \ldots, k_1 - 1\} & k_{m+1} < k_1 \\ k_{m+1} - 1 \in \{k_1, \ldots, n\} & k_{m+1} > k_1 \end{cases} \tag{S.44}$$

is a sequence of n distinct column indices, such that the factor corresponding to the l_mth column possesses at least one item of factor complexity one w.r.t. $\tilde{\Lambda}^{(m)} = \tilde{\Lambda}|_{\{l_m,\ldots,l_n\}} = \Lambda|_{\{k_{m+1},\ldots,k_{n+1}\}} = \Lambda^{(m+1)}$ for $m \in \{1, \ldots, n\}$.

Therefore, $\tilde{\Lambda} \in \mathbb{R}^{m \times n}$ is the loading matrix of a reduced model with n columns. If Λ is the loading matrix of a conditionally respectively unconditionally reduced model, then the same holds true for $\tilde{\Lambda}$ as well.

By the induction hypothesis, $\tilde{\Lambda}$ has full rank for all but a null set of parameter values if it is the loading matrix of a conditionally reduced model and $\tilde{\Lambda}$ has full rank for all parameter values if it is the loading matrix of an unconditionally reduced model.

It follows that $\operatorname{rank} \Lambda = \operatorname{rank} \tilde{\Lambda} + \operatorname{rank} \boldsymbol{\lambda}_{k_1} = n + 1$ for almost all but a null set of parameter values if the model is conditionally reduced and all parameter values if the model is unconditionally reduced, which concludes the induction step. ■

The subsequent corollary specifies the relation between unconditionally vs. conditionally reduced models and global identification vs. generic global identification.

Corollary S.12. *In the setting of Theorem S.3, a composed model with linear constraints on the cross-model factor covariances and generically globally identified reduced primary models is generically globally identified.*

Moreover, if the primary models are unconditionally reduced models and globally identified everywhere, then the composed model is globally identified everywhere.

Proof. From Lemma S.11 it follows that the primary models have full rank for at least almost all parameter values. Thus, the composed model is generically globally identified.

By Lemma S.11, if the primary models are unconditionally reduced primary models, then their loading matrices have full rank for all parameter values. Thus, if the primary models are globally identified everywhere, then the composed is globally identified everywhere. ■

References

Bekker, P. A., & ten Berge, J. M. F. (1997). Generic global indentification in factor analysis. *Linear Algebra and its Applications*, *264*, 255–263. https://doi.org/10.1016/s0024-3795(96)00363-1

Bollen, K. A. (1989). *Structural equations with latent variables.* John Wiley & Sons. https://doi.org/10.1002/9781118619179

Bourbaki, N. (1989). *Algebra I: Chapters 1–3.* Springer.

Horn, R. A., & Johnson, C. R. (1991). *Topics in matrix analysis.* Cambridge University Press. https://doi.org/10.1017/cbo9780511840371

Horn, R. A., & Johnson, C. R. (2013). *Matrix analysis* (2nd ed.). Cambridge University Press.

Jöreskog, K. G. (1978). Structural analysis of covariance and correlation matrices. *Psychometrika*, *43*(4), 443–477. https://doi.org/10.1007/bf02293808

Kenny, D. A., & Milan, S. (2012). Identification: A non-technical discussion of a technical issue. In R. H. Hoyle (Ed.), *Handbook of structural equation modeling* (1st ed., pp. 145–163). The Guilford Press.

Lang, S. (1987). *Linear algebra* (3rd ed.). Springer. https://doi.org/10.1007/978-1-4757-1949-9
